# Impacts of the Catalyst Structures on CO_2_ Activation on Catalyst Surfaces

**DOI:** 10.3390/nano11123265

**Published:** 2021-11-30

**Authors:** Ubong J. Etim, Chenchen Zhang, Ziyi Zhong

**Affiliations:** 1 Department of Chemical Engineering, Guangdong Technion-Israel Institute of Technology (GTIIT), Shantou 515063, China; ubong.etim@gtiit.edu.cn (U.J.E.); chenchen.zhang@gtiit.edu.cn (C.Z.); 2 Wolfson Faculty of Chemical Engineering, Technion-Israel Institute of Technology (IIT), Haifa 32000, Israel

**Keywords:** CO_2_ conversion, CO_2_ activation, metal oxide nanoparticles, oxygen vacancies

## Abstract

Utilizing CO_2_ as a sustainable carbon source to form valuable products requires activating it by active sites on catalyst surfaces. These active sites are usually in or below the nanometer scale. Some metals and metal oxides can catalyze the CO_2_ transformation reactions. On metal oxide-based catalysts, CO_2_ transformations are promoted significantly in the presence of surface oxygen vacancies or surface defect sites. Electrons transferable to the neutral CO_2_ molecule can be enriched on oxygen vacancies, which can also act as CO_2_ adsorption sites. CO_2_ activation is also possible without necessarily transferring electrons by tailoring catalytic sites that promote interactions at an appropriate energy level alignment of the catalyst and CO_2_ molecule. This review discusses CO_2_ activation on various catalysts, particularly the impacts of various structural factors, such as oxygen vacancies, on CO_2_ activation.

## 1. Introduction

The presence of CO_2_ in high concentrations in the atmosphere leads to a myriad of harmful consequences, including climate change and sea-level rise. Therefore, CO_2_ emission mitigation has become a top priority of various governments globally, and research interest in recent years is being dedicated to deriving the positive impact of the CO_2_ levels through efficient capture and utilization. An economically viable approach to mitigating the monstrous effect of CO_2_ and reducing its presence in the environment is to transform it into value-added products. The conversion of CO_2_ to fuels and chemicals has attracted increasing research attention globally, and great achievements have been recorded in the production of C_1_ products, such as methanol, methane, and formic acid [1,2,3].

CO_2_ is a chemically inert molecule due to its kinetically stable nature; thus, its conversion by reduction to economically viable products relies on its activation to kinetically vibrant species [4]. The stable nature of CO_2_ and high activation barrier (1.9 eV) implies that its transformation over catalyst surfaces should create a unique environment for facilitating activation pathways [5,6,7,8,9]. It is possible to apply either homogeneous or heterogeneous catalysis to transform CO_2_ into value-added products, the latter through thermo-, electro-, or photocatalysis, to induce the catalytic reactions [10,11]. Each of these processes comes with its shortcomings and benefits. The electro/photocatalytic processes have advantages in tuning the reaction products but are difficult to scale up [12]. Given the intricacy of the accompanying reaction mechanisms and the dynamics of catalysis under reaction conditions, particularly for the heterogeneous system, there is still a lack of very fundamental understanding of the chemistry of CO_2_ reduction. In the presence of small but reactive molecules such as hydrogen or water, CO_2_ can be transformed into stable products over heterogeneous catalysts with more favorable thermodynamics. The catalyst must possess activity for activating CO_2_ in addition to enabling effective conversion reactions. Adsorption and activation are two important steps that occur during the CO_2_ reduction on catalyst surfaces. The adsorption (physisorption and chemisorption) of CO_2_ on the surface of heterogeneous catalysts has become a subject of growing research interest in recent years [13].

The activation of CO_2_ on the heterogeneous catalyst surface yields CO_2_-derived species that eventually convert to useful products. Several CO_2_-derived species, including carboxylate and carbonates, have been identified, irrespective of which approach is adopted for CO_2_ conversion [6]. The generation of specific activated carbon species determines the kind and selectivity of the product(s). Co-reactants such as water contribute to the overall reduction reaction by promoting adsorption and subsequent activation. As with most chemical conversion technologies, the choice of catalyst is also an important factor, which must have suitable selectivity and activity for activating CO_2_ under relatively mild conditions. Many studies reported in the literature tried to understand how to promote the chemisorption and subsequent activation of CO_2_ by focusing on catalysts preparation and structures. Specifically engineered or surface-modified catalysts have shown improved performance for CO_2_ activation. Catalysts (metal oxides) with rich surface defects or high concentrations of oxygen vacancies have been particularly effective for CO_2_ activation. Oxygen vacancies can greatly influence the interaction of CO_2_ with the surface and enhance the adsorption of CO_2_ molecules. Thus, oxygen vacancies play important roles in CO_2_ conversion. Surface defects can be created in metal oxide catalysts either during reactions or incorporated by external methods. Understanding the nature of CO_2_ in the activated form and the catalyst characteristics will be important in developing novel strategies for activating CO_2_. An appreciable understanding of the reaction of CO_2_ on pure metal surfaces is well documented; however, less is known about the reactivity of CO_2_ on metal oxide surfaces, e.g., how the surface defects contribute to the mechanism of CO_2_ activation. The central aim of this work is to discuss the CO_2_ activation mechanism on metal oxide surfaces, particularly the roles of the surface defects in the activation process.

### 1.1. CO_2_ Conversion to Chemicals: Challenges, Thermodynamics, and Kinetics

The development of environmentally viable technologies for the utilization of CO_2_ as a chemical feedstock remains a great challenge because of its thermodynamic stability (CO_2_ possesses a highly stable π-conjugated structure) and kinetic inertness. Regarding the energy requirement, reactions involved in CO_2_ conversion can be classified into two major categories: The first category involves adding CO_2_ to a reaction where the other reactants with higher Gibbs free energy supply energy; such reactions produce the likes of carboxylates and lactones, carbamates, urea, isocyanates, and carbonates. These reactions are energetically favorable and often can be run without any catalyst. The second category is the CO_2_ reduction reaction, through which important industrial chemicals such as formate, oxalates, carbon monoxide, formaldehyde, methanol, and methane can be obtained. Table 1 presents an overview of the standard enthalpies (ΔH°_298K_) and Gibbs free energies (ΔG°_298K_) for different products formed from CO_2_ conversion reactions. These reactions require an external energy source to activate the strong C=O bond in CO_2_ and a catalyst to lower the energy barrier. It will be energy-demanding if CO_2_ is used as a single reactant (Table 1, Equation (1)). However, it becomes thermodynamically easier if another substrate molecule, such as H_2_, with higher Gibbs free energy, is introduced as a co-reactant (Table 1, Equations (2)–(10)). Another difficulty is that the final products derived from CO_2_ are mostly in the liquid phase (e.g., formic acid, methanol, and higher hydrocarbons). As these reactions involve a phase change from gaseous reactants into liquid products, they are thus entropically unfavorable.

The unfavorable thermodynamic character of CO_2_ stems from its highly stable chemical nature. Both ∆S and ∆H (−393.5 kJ·mol^−1^), which govern the thermodynamics, are unfavorable for CO_2_ conversion as a result of the very strong C=O bond with a higher dissociation energy (~750 kJ·mol^−1^) [14,15]. Thus, highly active catalysts are necessary for the activation of CO_2_. Good catalysts should be able to break the kinetic barriers and enable the chemical conversion of CO_2_ under relatively mild reaction conditions [16]. While efforts have been demonstrated in this regard, leading to fair knowledge of the reaction mechanisms, more research inputs are still needed to comprehend the principles governing the catalytic transformation of CO_2_ into value-added chemicals that can drive the industrial scale utilization.

### 1.2. General Properties of CO_2_ Molecule: Molecular Structure and Bonding Properties

**Figure 1:**
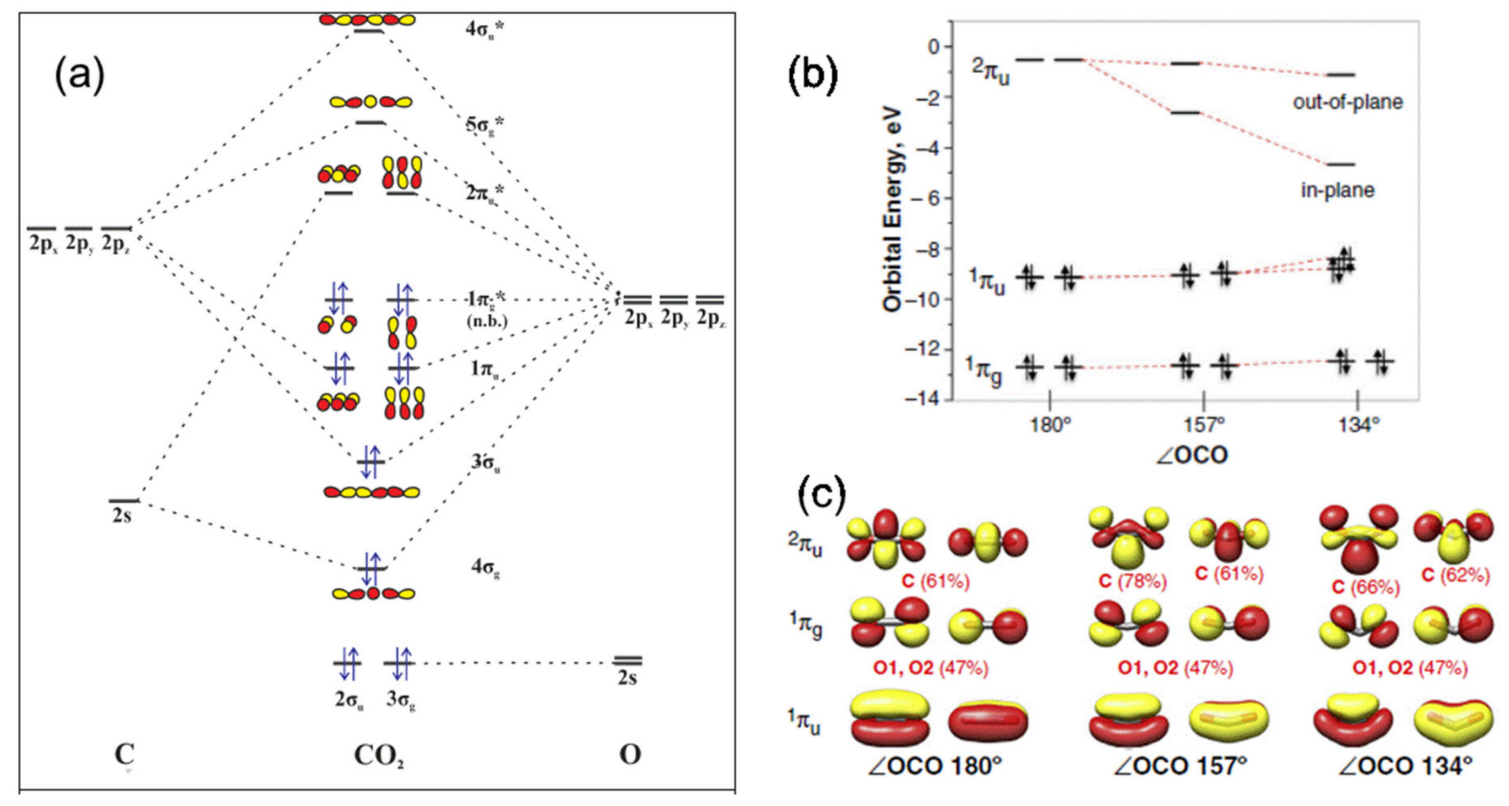
(**a**) Molecular orbital of CO_2_ at the ground state. Reproduced with permission from the authors of [23]. Copyright 2016 Elsevier B.V. (**b**) Walsh diagram for the orbital energies upon CO_2_ bending, and (**c**) molecular orbitals of CO_2_ at different OCO angles (carbon and oxygen weights are given in %). Reproduced with permission from the authors of [25]. Copyright 2015 Elsevier B.V.

CO_2_ is a linear non-polar molecule in its ground state possessing two equivalent C–O double bonds, and its main properties are summarized in Table 2 [18,19,20,21]. Its high oxidation state of carbon (+4) makes it a thermodynamically stable molecule, requiring a high energy input for activation and reaction for producing value-added products. The activation of CO_2_ typically involves altering the molecular properties, such as the C–O bond length and O–C–O angle. According to the molecular orbital diagram of CO_2_ (Figure 1a), there are a total of 16 valence electrons distributed among the three atoms (one carbon and two oxygen atoms) in the molecule. The carbon atom exhibits an electrophilic character, conveying an exception to the overall energetics of the CO_2_ molecule, and thus the nucleophilic and electrophilic reactions are preferred on the C- and O-atoms, respectively. In other words, activation of CO_2_ can be achieved both nucleophilically and/or electrophilically through the carbon or oxygen atom, respectively [22]. For example, nucleophilic compounds such as water and hydroxides prefer to react at the carbon center; the carbon atom could also gain electrons from a catalyst surface. On the other hand, oxygen atoms in the CO_2_ molecule with lone pairs of electrons can donate them; thus, CO_2_ molecules can simultaneously donate or accept electrons, leading to mixed coordination (i.e., binding on both C and O atoms). In the presence of a substrate such as water, the charge on the carbon atom can increase according to a DFT study using a polarizable continuum model with a linear geometry [7], which can boost the carbon atom reactivity of CO_2_. Non-bonding orbitals are located on the 1s shells of the C and O atoms and do not participate in CO_2_ bonding [23,24]. The introduction of an electron into the antibonding orbital 2π_u_, as shown in Figure 1b, results in the formation of the CO_2_ radical anion (CO_2_^−^) or CO_2_^δ−^, which can be stabilized by changing the structure from the linear to the bent form [23]. For the anionic CO_2_ molecule, the carbon atom is nucleophilic, and the molecule has a bent geometry (Figure 1c).

## 2. CO_2_ Activation and Different Modes

CO_2_ is an inert molecule, but its reactivity can be increased by activation, which can be measured by the difference in molecular properties (geometry and electronic properties) of the charged species relative to those in the stable ground state [5]. The activation of a stable CO_2_ molecule can take one of the following modes:

- bending of the O–C–O angle from 180 degrees,
- elongation of at least one of the two C–O bonds,
- polarization of the charges on C and O, leading to the transfer of charge/electron to CO_2_,
- hydride transfer, or
- redistribution of charges.

Generally, over heterogeneous catalysts, the activation of CO_2_ molecule involves a charge transfer (effect iii) from the catalyst to the molecule, which results in elongation of the C–O bond length and reduction in/bending of O–C–O bond angle (effects i and ii). The activation can proceed in the presence or absence of reducing species such as hydrogen [26,27]. Mondal et al. [27], via ab initio DFT calculations, observed the C–O bond in CO_2_ to increase from 1.16 Å to between 1.27 and 1.42 Å on Zr clusters. Furthermore, the O–C–O bond angle changed from 180 to between 115 and 136°. These occurred through chemisorption and subsequent activation of the free CO_2_ on the surface of the metal cluster as a result of charge migration from the Zr cluster to the CO_2_, forming partially charged CO_2_^δ−^ species.

At the molecular level, the CO_2_ activation results from partial electron transfer into the LUMO [28]. Based on the molecular orbitals analysis, the reverse charge donation to LUMO of the activated CO_2_ is responsible for the CO_2_ binding, which determines the degree of CO_2_ activation. Consequently, the binding energy originating from the backward donation can be explored to explain the degree of CO_2_ activation [29]. However, the binding energy is affected by the coordination number and the deformation of the ligand. Definitive evidence for CO_2_ activation is the bending of the linear OCO configuration. The more bent the geometry, the lower the energy level of the in-plane (i.e., to the plane of bending) contribution of the 2π_u_ LUMO will be.

CO_2_ bending lowers the LUMO’s energy to a significant degree, particularly that of the in-plane 2π_u_ orbital (Figure 1b), and increases the carbon electron density associated with it, facilitating the transfer of an electron to the molecule. As a result, bending leads to the C–O bond weakening by stretching up to 0.11 Å in comparison with the linear state. This may lead to the dissociation of CO_2_ on the catalyst surface into CO and O species. Unlike the HOMO that possess the characteristics of the O p-orbital, the LUMOs exhibit mainly the C p-orbital character. These features enhance the CO_2_ reduction (electron accepting) ability.

However, some of the above indicators or differences in other properties may not sufficiently explain the activation of the CO_2_ molecule. It has been shown that bending CO_2_ molecules to smaller O–C–O angles (less than 180 degrees) and an electron transfer to the π*-antibonding orbital correlate more with strong adsorption, leading to the formation of undesired carbon species such as carbonates [30]. CO_2_ activation on metal oxide surface as studied using an Artificial Intelligence (AI) model revealed the binding of an O atom to a surface cation, resulting in an increase in the C–O bond length and weakening of the bond strength. This confirms only the C–O bond elongation as a valid indicator of CO_2_ activation [30].

**Figure 2:**
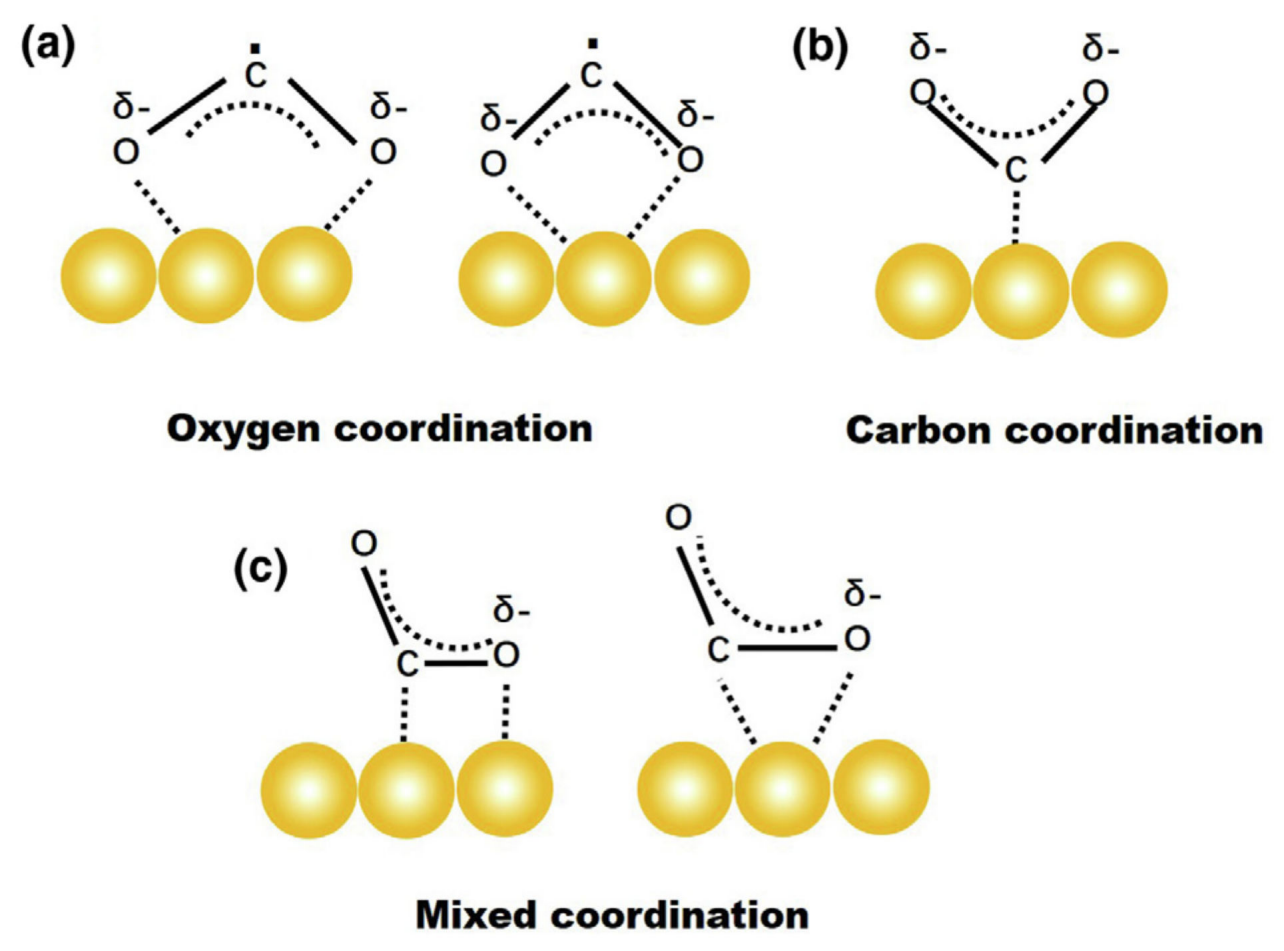
Possible structures of CO_2_^δ−^ on heterogeneous catalyst surface: (**a**) oxygen coordination, (**b**) carbon coordination and (**c**) mixed coordination. Reproduced with permission from the authors of [31]. Copyright 2016 The Royal Society of Chemistry.

The transfer of electrons to CO_2_ always precedes a key step—adsorption (chemisorption or physisorption), and the structural properties of the activated CO_2_ species are evident after adsorption of the CO_2_ molecule. In many examples, the interaction that results upon chemisorption leads to the formation of CO_2_^δ−^. This carbon species present different structures depending on the mode of adsorption [31,32], including oxygen coordination, carbon coordination, and mixed coordination (Figure 2). Coordination through the oxygen atom results in two possible structures of bidentate species as shown in Figure 2a. The formation of a carbonate-like species is evident in the carbon binding mode. Structures of both species can be observed in the combined coordination mode. These different binding modes play a crucial part in determining the reaction pathways as a result of the different possible intermediates that can form [32].

The other chemisorbed CO_2_ species is the CO_3_^2^^−^, which is direct evidence of the transfer of charge from surface oxygen to the CO_2_ and a molecule bending to form a [O–CO_2_]^2^^−^ complexes [33].

The chemisorption of CO_2_ is a critical step in CO_2_ conversion reactions [34,35,36]. For example, the dissociation into CO and O or hydrogenation to form different products such as methane or methanol follows the adsorption of CO_2_. However, the adsorption process must avoid producing inactive surface species (e.g., carbonates) that poison catalysts [26]. From both experimental and theoretical investigations, the activation of CO_2_ over a heterogeneous catalyst surface involves its adsorption followed by charge transfer (electron) from the catalyst active site to the CO_2_ molecule [35,37,38]. Upon accepting an extra electron, the molecular CO_2_ forms the radical anion, CO_2_^−^ or CO_2_^δ−^ [31]. This perturbation of the molecular state results in weakening of the C–O bond and reduction in O–C–O bond angle (bending) [34,39]. CO_2_ activation was observed from the infrared vibrational spectra of the CO_2_ molecule adsorbed onto Ti_8_C_12_ [35]. A peak that was obviously lacking in the gaseous state CO_2_ molecule was observed. CO_2_ is adsorbed and activated by dissociating into CO and O. In some CO_2_ transformation reaction mechanisms, CO has been identified as an important intermediate in the reaction pathway.

**Scheme 1:**
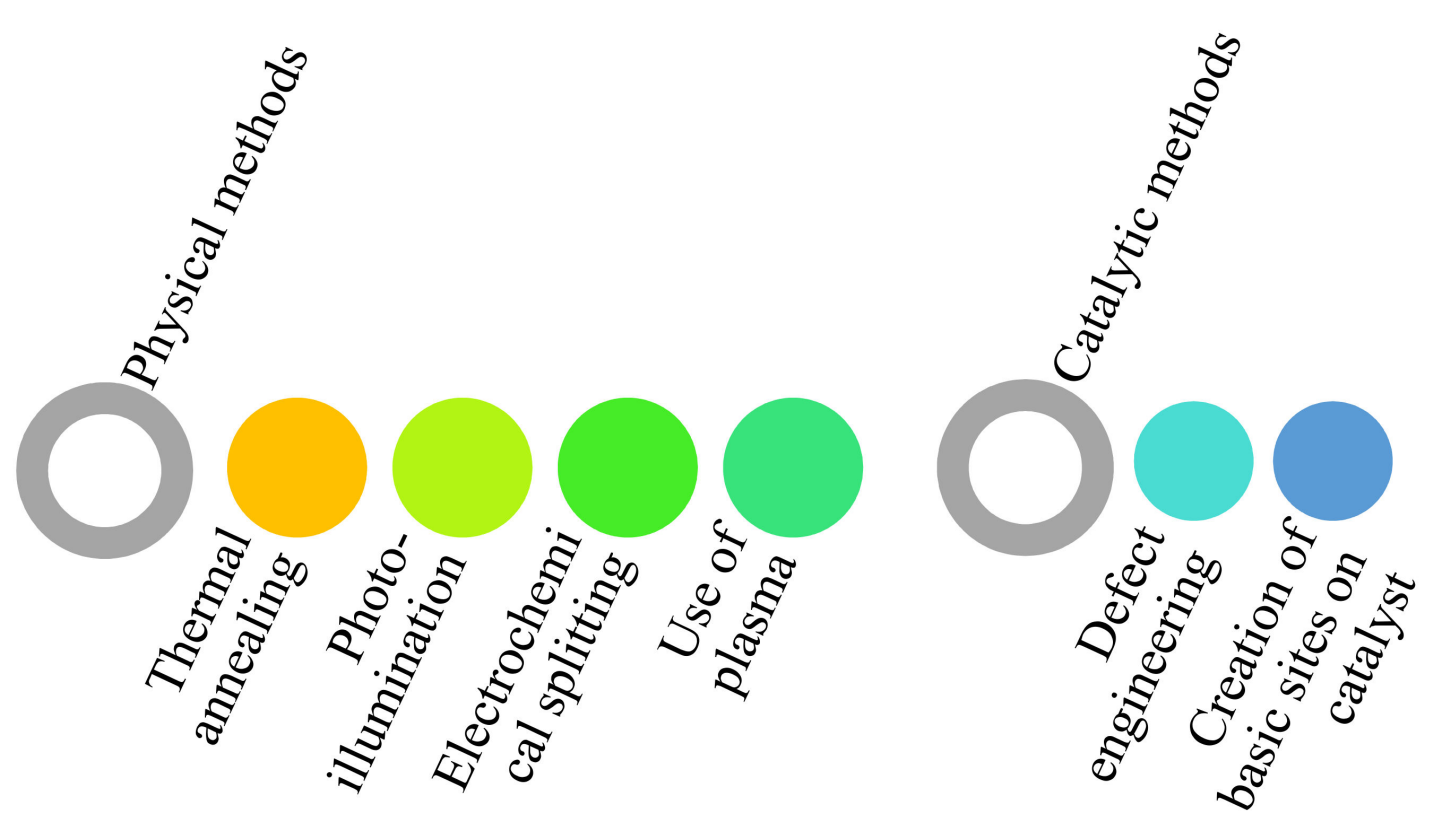
Strategies for enhancing CO_2_ activation of oxide-based catalyst surface.

The adsorption and activation of CO_2_ molecules can be enhanced by adopting the strategies presented in Scheme 1.

### 2.1. Photocatalytic Activation of CO_2_

Solar-driven catalytic (photo)reduction of CO_2_ with H_2_O at low temperature (room temperature) and pressure (atmospheric pressure) is an approach for economically utilizing large amounts of CO_2_ emitted [40]. CO_2_ photoreduction mainly involves the following elementary steps:

- Photon absorption and excited carrier generation.
- Activation of CO_2_ to form an anion radical, CO_2_^•^^−^, or other intermediates by the photoexcited electrons.
- Dissociation of the C–O bond, involving the participation of protons and electron transfer, generating different products.
- Desorption of reduced products from the active sites [41,42].

During the photoreduction process in the presence of a photocatalyst, an electron–hole pair is generated upon photo-illumination of the catalyst surface. The generated electrons transfer to the stable CO_2_ molecule and activate it to intermediates species that can be much easily converted to a variety of chemical and fuel products, including CO, CH_4_, CH_3_OH, and HCOOH. However, water, which is typically used as the reducing agent in CO_2_ photoreduction reaction, results in low photocatalytic efficiency [40,43]. Some of the reasons for the observed low efficiency on TiO_2_ photocatalyst include:

- the one-electron transfer to form CO_2_^−^ is thermodynamically unfavorable as it requires a very negative reduction potential of −1.9 V NHE;
- the photoexcited holes (or OH radicals) easily oxidize water to oxygen, or oxidize the intermediates and products converted from CO_2_ to undesirable products (reverse reactions); and
- the electron–hole pairs recombination rate is much faster.

Almost no photocatalyst can provide sufficiently high energy to transfer a single photoexcited electron to a neutral state CO_2_. This remains the most important obstacle to the photoreduction of CO_2_. Consequently, the multielectron process is often adopted; however, this leads to slow reaction kinetics. The above limitations can be checked by stabilizing the LUMO of CO_2_ close to or lower than the conduction band minimum (CBM) of the semiconductor; this especially can decrease the strongly negative potential [44]. In the photoreduction process, it is possible to regulate light irradiation to tune the CO_2_ conversion as desired in comparison with the conventional thermal reaction. Thus, the photo-assisted heterogeneous catalytic reaction can proceed with quite low energy consumption for product formation and a much faster response for the changes in the reaction condition.

The photoreduction of CO_2_ also involves important steps, namely, adsorption, activation of CO_2_, and dissociation of the C=O bond. Although the adsorption precedes the activation step followed by the dissociation step, they all work synchronously to realize the overall process, and these steps are often paid less attention in the literature. The transfer of charge (photogenerated electrons) to the CO_2_ molecule from the photocatalyst surface occurs during the activation step. At the metal-oxide interface, the ease of electron transfer depends on the electron affinity of CO_2_ and its interaction with the catalyst surface [45]. In addition, the adsorption and activation properties of CO_2_ on the surface of photocatalyst greatly affect the activity and product selectivity. Generally, the activation and subsequent reduction of CO_2_ involves the participation of protons and electrons transfer (single or multiple electrons). This generates CO_2_^−^ by one-electron transfer to CO_2_, which is an important activated intermediate in CO_2_ reduction reactions [40]. A more feasible pathway involves multiple electrons and a corresponding number of protons. Therefore, controlling the transfer of electrons and protons is crucial for the activation of CO_2_ [46]. Understanding the CO_2_ activation mechanism is essential to guide the improvement and development of effective photocatalysts to promote CO_2_ reduction efficiency [47]. We herein briefly discuss the activation of CO_2_ through the photocatalytic reduction mechanism. The adsorption of CO_2_ on photocatalysts through interactions with surface atoms can activate CO_2_ by transferring an electron from excited photocatalysts to the CO_2_, although adsorption and the interactions between CO_2_ and the photocatalyst surface sometimes are not strong enough to sufficiently lower the barrier and enable the transfer of conduction band electrons. In this case, efforts are directed at improving the photocatalytic efficiency by fabricating photocatalysts with various features to enhance the chemisorption of CO_2_ [46].

Researchers have studied many strategies to promote adsorption and activation of CO_2_ on metal oxides. Both theoretical and experimental studies have revealed that these processes are significantly influenced by the crystal phase of TiO_2_ photocatalysts and their surface defects [48,49,50,51]. Graphene-laden catalysts have been observed with high photocatalytic activity. In addition to increasing CO_2_ adsorption, there are reports about the increasing separation efficiency of photogenerated electrons and holes of graphene containing photocatalysts. Reduced graphene (rGO)-adorned Pt/TiO_2_ photocatalysts with enhanced photogenerated charges separation and CO_2_ adsorption was reported by Zhao et al. [52]. The surface hydroxyl and abundant *p* electron of rGO provide adsorption and activation sites for CO_2_. Doping photocatalysts with alkaline-earth or transition metal oxides can provide more surface basic sites for CO_2_ adsorption and activation, thus improving reaction kinetics and product selectivity [53,54]. For example, 1.0 wt% MgO–Pt–TiO_2_ gave a superior photocatalyst performance in reducing CO_2_ into CH_4_ in the presence of H_2_O in comparison with Pt–TiO_2_ [55]. MgO presence provided more surface basic sites for chemisorption of CO_2_. Insertion of defects or vacancies in photocatalysts can provide more sites for CO_2_ adsorption and activation. Zhao et al. [56] constructed O vacancies in ZnAl-LDH nanosheets for photocatalytic reduction of CO_2_. The resulting Zn^+^–V_O_ complexes served as trapping sites for adsorption of CO_2_ and H_2_O that boosted the photocatalytic CO_2_ reduction activity. Incorporating an appropriate co-catalyst can enhance CO_2_ activation. Pt/Cu_2_O-modified TiO_2_ exhibited high activity for the selective photocatalytic reduction of CO_2_ into CH_4_. Deposited Pt can capture photogenerated electrons and generate H_2_ and CH_4_, while the Cu_2_O can enhance the CO_2_ chemisorption and inhibit H_2_O [57]. In general, on the surface of photocatalysts, these strategies can be adopted to activate CO_2_ molecules essential for CO_2_ conversion. Photocorrosion of oxide photocatalysts can generate in situ photoinduced oxygen vacancies to activate a defect reaction for splitting CO_2_ under mild reaction conditions that eliminate the need for high temperature and water presence. Wang et al. [58] used an amorphous semiconductor photocatalyst with weak lattice constraint via the photocorrosion mechanism to facilitate the generation of oxygen vacancies, which can react with oxygen atoms of CO_2_, splitting it into C and O_2_. Such a defect reaction can be sustained by continuous photogenerated hole oxidation of surface oxygen atoms of the photocatalyst to form an oxygen vacancy and O_2_. On the other hand, the photogenerated electrons would reduce the carbon species of CO_2_ to solid carbon. A major challenge is how to generate a high concentration of oxygen vacancies on the photocatalyst surface [58].

**Figure 3:**
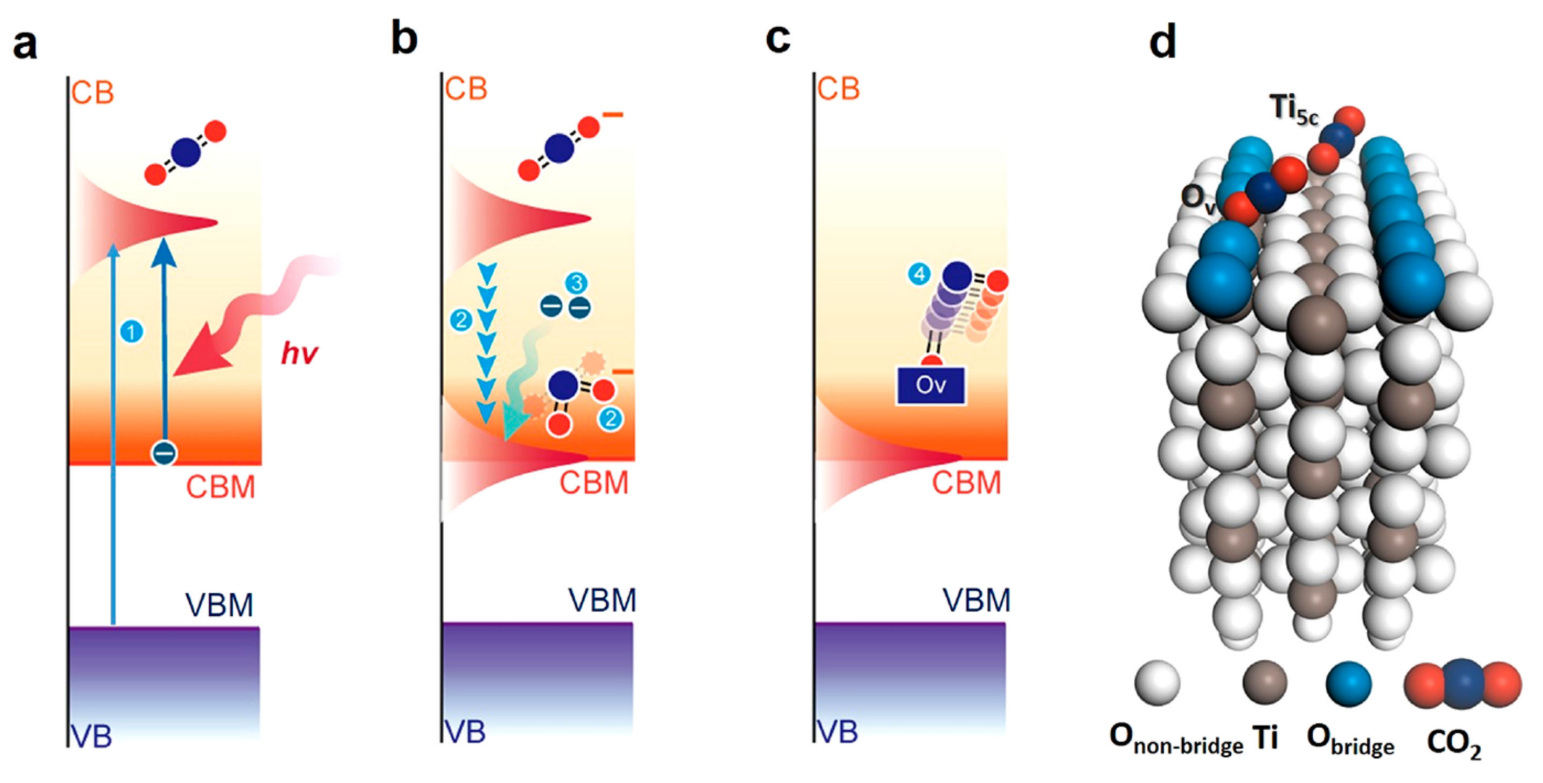
CO_2_ photoactivation on the TiO_2_ surface (**a**–**c**). CB: conduction band; CBM: conduction band minimum. Schematic of the CO_2_ molecule adsorbed in the oxygen vacant site and Ti_5C_ sites on the TiO_2_ (110) surface (**d**). Reproduced with permission from the authors of [44]. Copyright 2020 American Chemical Society.

Recently, photoinduced electrons have been shown to benefit the activation of stable CO_2_ molecules for the photoreduction process [44,59]. Hot electrons generated upon light irradiation enhanced the CO_2_ conversion. The enhancement was related to the change in the HOMO–LUMO energy gap of the CO_2_ adsorbed on the metal surface. According to DFT calculations, CO_2_ can strongly bind to Ru surface, and the energy bandgap is reduced from 8.5 eV to 2.4 eV in free CO_2_ and CO_2_ adsorbed to the Ru surface, respectively. The CO_2_ dissociation into CO is promoted with light irradiation because it lowers the reaction energy for CO_2_ reduction. Total energy consumption of 37% was required for the light-irradiated process compared to the case without light irradiation at a CO_2_ conversion rate of 15% [59]. On the rutile TiO_2_ (110) surface investigated theoretically, photo-excited electron induced CO_2_ reduction through a process that involves the formation of a transient CO_2_^•^^−^ with bent geometry through the photoexcitation of one electron to the LUMO of CO_2_ as shown in Figure 3, and summarized as follows: (1) photoexcitation generates CO_2_^•^^−^, (2) transient CO_2_^•^^−^ excites the bending and antisymmetric stretching vibrations that stabilize the CO_2_ LUMO, (3) CO_2_ traps hot electrons and forms a new CO_2_^•^^−^, and (4) CO_2_^•^^−^ dissociates on oxygen vacancy [44]. The excitation of the bending and antisymmetric stretching vibrations can sufficiently decrease the energy of the CO_2_ LUMO to below that of the CBM of TiO_2_. Although this was demonstrated with TiO_2_, it is believed that conclusion can be widely applied to other metal oxides as well as to other semiconductors.

### 2.2. Electrocatalytic Activation of CO_2_

Electroreduction of CO_2_ is another appealing approach for CO_2_ valorization. The principles of operation, striking features, and the challenges of this process have been discussed in the literature [10,12,60,61,62]. The electrochemical CO_2_ reduction is a multielectron and multiproton process involving several transfer pathways that result in different products [63], and this is of immense advantage over the single electron process in terms of electrochemical potential requirements [62]. As in the thermo- and photo-catalytic processes, the electrocatalytic reduction involves the adsorption and activation of CO_2_ on the catalyst surface to product precursors. Following adsorption of CO_2_ on the electrocatalytic surface is the electron transfer and/or proton migration that initiates the C=O bond cleavage and subsequent formation of C–O and/or C–H bonds [64]. The adsorbed product species then desorb and diffuse from the catalyst surface. From both theoretical and experimental studies, the adsorption and activation steps are the most critical of the CO_2_ electroreduction process not only due to the high thermodynamic stability of the neutral CO_2_ molecule, but also the requirement of a large amount of energy for breaking the kinetic barrier [62], considering the strongly negative redox potential (−1.90 V) compared with SHE [65]. As the rate-determining step, the chemisorption and subsequent activation of CO_2_ on the electrocatalyst surface would form CO_2_^•−^ intermediate that can further be reduced to the *CO, *COH, *CH_2_ product precursors, or effect C–C coupling to form multicarbon products [65,66,67,68]. Consequently, understanding the CO_2_ activation mechanisms over electrocatalysts is necessary for a proper comprehension of the electroreduction transformation to different products, and for improving its performance. To this end, researchers have focused on how to achieve efficient CO_2_ activation on electrocatalysts. This includes electrocatalyst engineering and surface modification [69]. Such catalysts need to be tailored with electron-donating centers for the efficient chemisorption of CO_2_ molecules.

The proper design of the electrocatalyst active phase can improve the activation of the neutral CO_2_ molecule. Xiao et al. [69] conducted a detailed study of the CO_2_ adsorption on Cu electrocatalyst employing different catalyst models. It was found that only the catalyst with a combination of surface Cu^0^/Cu^+^ sites promoted CO_2_ activation, favoring kinetics and thermodynamics. The Cu^0^ sites bind to activated CO_2_, whereas Cu^+^ sites dilute the negative charge by binding to water molecules in the electrolyte solution. This translated into the observed increase in the onset potential and peak Faradaic efficiency for CO production [69].

The combination of experiments and theoretical calculations on the surface and electronic structure of Cu electrocatalyst revealed that the presence of a thin suboxide species below the Cu surface could bind the CO_2_ in the physisorbed configuration at 298 K; in the presence of H_2_O, this suboxide is essential for further converting to the chemisorbed CO_2_ and subsequently toward CO_2_ reduction products such as formate and CO [70]. Indeed, it has been suggested that the CO_2_ might dissociate more easily on pre-oxidized Cu surfaces [71], but evidence to support this is scarce. The results of the CO_2_ reduction to CO with annealed Cu_2_O revealed a linear Tafel plot over the range of overpotentials from 0.05 to 0.3 V with a slope of 116 mV/decade. The slope of this plot was in agreement with the rate-determining initial electron transfer to CO_2_ to form a surface adsorbed CO_2_^•^^−^ intermediate. The difference in Faradic Efficiency (FE) between the reduced Cu electrode and that of the polycrystalline Cu suggested that the Cu surface formed by reducing thick Cu_2_O layers could form the CO_2_^•^^−^ intermediate [71]. This presents the need for understanding the surface structures of electrocatalytic Cu particles for the CO_2_ activation and subsequent reduction to different products.

**Figure 4:**
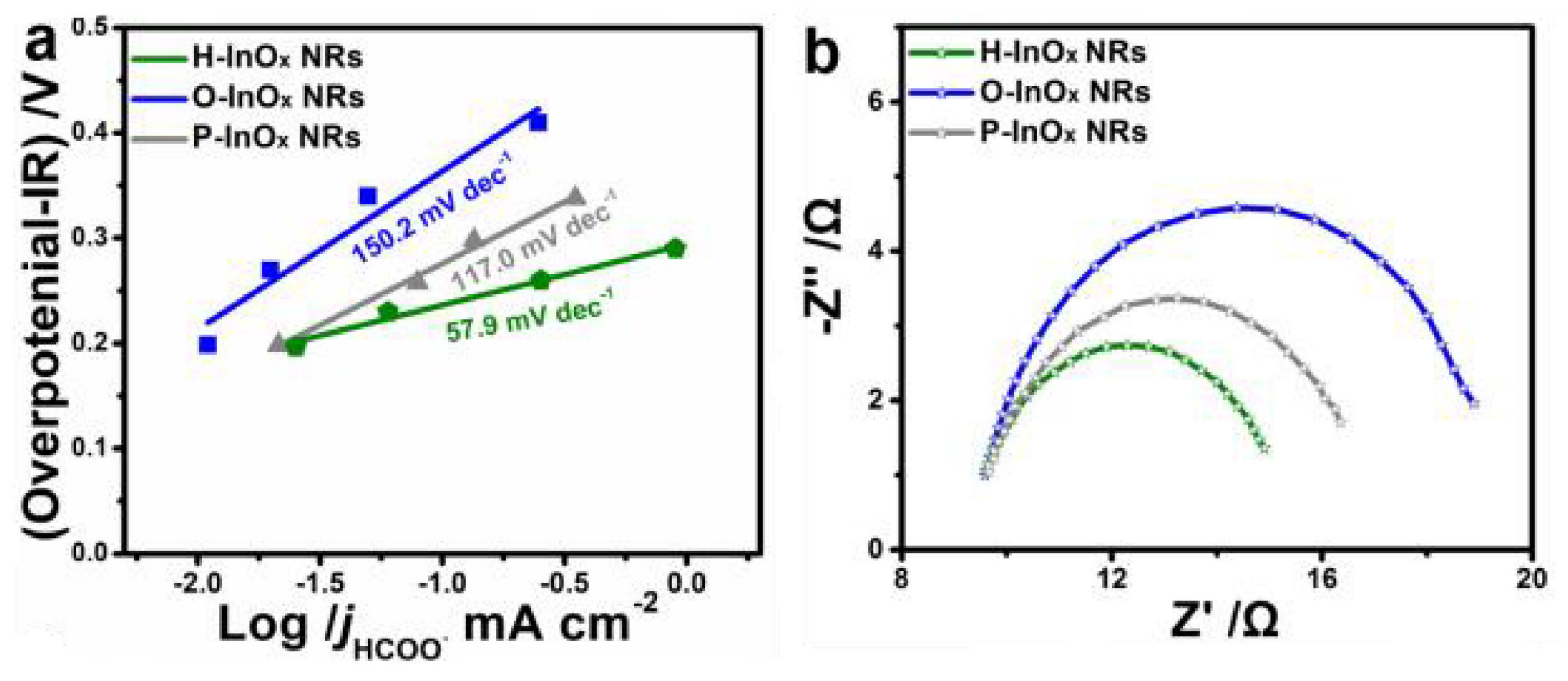
(**a**) Tafel plots and (**b**) Nyquist plots of HCOO^−^ for the electrocatalytic reduction of CO_2_ on InO_x_ catalysts. Reproduced with permission from the authors of [72]. Copyright 2019 John Wiley & Sons, Inc.

Engineering oxygen vacancy/surface defects in CO_2_-reduction catalysts has proven an indispensable method for generating and transferring surface electrons to CO_2_ molecules [72,73,74]. Gu et al. [73] reduced copper oxide nanodendrites with rich surface oxygen vacancies and obtained partially reduced Cu oxide catalyst with Lewis base sites for enhanced CO_2_ adsorption and subsequent electroreduction. Furthermore, strong binding of *CO and *COH intermediates to CuO*_x_*surface was observed. Theoretical calculations and catalytic test results showed that oxygen vacancy can provide a surface for binding, and as a result, the partially reduced CuO*_x_*nanodendrites had a high electrocatalytic activity for C_2_H_4_ production. ZnO nanosheets rich in oxygen vacancies exhibited a current density of −16.1 mA cm^−2^ with a FE of 83% for CO production at −1.1 V versus RHE in the CO_2_ electrochemical reduction [74]. According to DFT calculations, introducing oxygen vacancies increased the charge density of ZnO around the valence band maximum, resulting in the enhanced activation of CO_2_. In addition, mechanistic studies revealed that introducing oxygen vacancies into ZnO nanosheets increased the binding strength of CO_2_ and facilitated CO_2_ activation [74]. The electrochemical properties of InO_x_ electrocatalysts (P-InO_x_ NRs, O-InO_x_ NRs, and H-InO_x_ NRs) in CO_2_-saturated NaHCO_3_ solution were investigated. It was found that H-InO_x_ NRs exhibited the best performance, generating a current density of 26.6 mA·cm^−2^ at −1.05 V versus RHE. This catalyst had the highest oxygen vacancy concentration compared with others. The current densities for HCOO^−^ at various overpotentials for the different InO_x_ catalysts are presented in Figure 4a. The Tafel plot of O-InO_x_ NRs, P-InO_x_ NRs, and H-InO_x_ NRs gave slopes of 150.2, 117.0, and 57.9 mV/decade, respectively, pointing to the different adsorption strength of the catalysts for CO_2_ molecules. H-InO_x_ yielding the least slope value indicates the pre-equilibrium transfer step (CO_2_ + H^+^ + e^−^ + * → HCOO*) rather than a chemical H^+^ transfer step as the rate-determining step [72]. The availability of hydroxyl groups on the surface of the electrocatalyst can enhance its adsorption properties. Huang et al. [64] functionalized the MOF surface with OH^−^ moieties and used it for the electrochemical reduction of CO_2_. They observed single-crystal to single-crystal structural transformation between the MOF and MOF-CO_2_, activating it to HCO_3_^−^, an important intermediate in the electrocatalytic reduction process [75]. Theoretical calculations revealed the formation of the HCO_3_^−^ species from the geometries of CO_2_ adsorption at both the O- and C-atoms. In addition, the OH-incorporated MOF sample exhibited high FE for CO, achieving approximately 100% at −0.6 V versus RHE, surpassing the 96% (−0.6 to −0.9 V) in most MOF-based catalysts [64].

### 2.3. Activation of CO_2_ by Homogeneous Catalysts

**Scheme 2:**
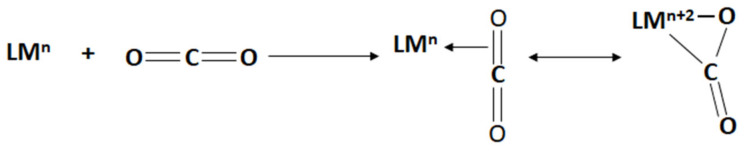
Activation of CO_2_ by transition metal complexes.

In an aqueous solution of transition metal salts, a CO_2_ molecule can be activated by forming a transition metal–CO_2_ complex via direct coordination [4,22,76,77,78]. This can be considered a combination of π electrons transfer to the metal and the oxidative addition of the C–O bond to the metal (Scheme 2) [22]. For a multi-metal complex, CO_2_ can also be activated by bridging between metals in the complex. The successful formation of the metal–CO_2_ complexes is key to CO_2_ activation. Upon forming the metal–CO_2_ bond, the activation energy for further reactions is sufficiently lowered, thus accelerating the reactions rates in various CO_2_ transformation processes. A number of factors, including the central metal itself, ligand set, and metal oxidation state, have been identified to affect the activation of CO_2_ on metal complexes [22].

Infrared spectroscopy (IR) has emerged as an excellent tool for the structural investigation of metal–CO_2_ interactions in complexes, such as M^+^(CO_2_)_n_ or M^−^(CO_2_)_n_ [79,80,81,82,83]. Review articles discussing the spectroscopic studies of the various types of CO_2_ interactions with metal complexes are available in the literature, which readers are directed to. A recent review on the subject was authored by Paparo and Okuda in 2017 [84].

We briefly discuss recent studies on the conversion of CO_2_ to chemicals and fuel via the formation of CO_2_-activated complexes. Catalytic reduction of CO_2_ to methanol and other products has been observed with transition metal complexes [22]; it is possible to develop highly active catalysts based on early transition metal complexes. It was found that anionic metal clusters (M_n_^−^) with 1, 2, and 6 atoms formed the activated complex (M_n_–CO_2_)^−^ for Cu, Ag, and Au [4]. The activation was a result of a strong interaction between the CO_2_ moiety and M_n_^−^ via the formation of a partial covalent M–C bond with a full delocalization of the electronic charge due to electron transfer from the HOMO of M_n_^−^ to the LUMO of CO_2_ as in metal–CO_2_ π-backbonding. This can easily be understood from the perspective of the orbital geometry and energetics following orbital mixing (orbital interaction between HOMO of M_n_^−^ and LUMO of CO_2_) [4,29].

## 3. Activation of CO_2_ on Heterogeneous Catalyst Surface

The interaction of CO_2_ molecules with metal-based surfaces has attracted intense research attention for a long time. Reduction of CO_2_ by H_2_ has been studied on some transition metals (e.g., Cu, Co, and Ni) and metal oxide catalysts (e.g., TiO_2_, CeO_2_, and In_2_O_3_). The catalytic surfaces for the activation of CO_2_ include metal sites, metal-oxide interfaces, and oxygen vacancies. For facile CO_2_ activation and conversion, efforts should be made to know, design, and optimize functional sites in heterogeneous catalysts, which involves constructing surface sites with a charge density gradient capable of reorganizing the electronic structures of CO_2_ and polarizing the adsorbed species [85].

Metal-based catalysts are capable of effectively activating the CO_2_ even in the absence of hydrogen [27]. These include pure metal surfaces, doped or promoted metal surfaces, and supported metal nanoparticles. On supported metal surfaces, CO_2_ activation occurs mainly through acceptance of charge from the metal; the oxide support plays a big part in the activation process through different mechanisms such as acid sites interactions and provision of oxygen vacant sites [86]. The metal surface is also important in the dissociation of H_2_ in CO_2_ hydrogenation reactions [6].

### 3.1. CO_2_ Activation on Representative Pure Metals

Metal nanoparticles sit on a metal oxide serve as active sites for the electron transfer. Transition metals such as Cu, Ni, and Fe are particularly energetic for activating CO_2_. Their surfaces possess high binding abilities to CO_2_. DFT calculations evidenced that on Pt, Rh, Ni, Cu, Ag, and Pd (111) surfaces, the affinity toward oxygen is different for the metals, which selects their reaction pathways for the CO_2_ activation in the RWGS) reaction. Pt, Ag, and Pd tend to favor the COOH-mediated mechanism, whereas Rh, Ni, and Cu dissociate CO_2_ into CO and O. This difference underlines the variation in the CO_2_ dissociation barrier of the different metal groups. Thus, the nature of interaction between the adsorbed O and the surface is critical for determining the CO_2_ dissociation barrier [87]. In the activation of CO_2_ via the charge transfer mode, which is the prevalent on metal surfaces, the partial and full charge transfer leads to the formation of CO_2_^δ−^ and CO_2_^−^, respectively. The degree of charge transfer can be analyzed on metal surfaces after adsorption. Physisorption and chemisorption of CO_2_ on single crystal surfaces of various metals have been studied by means of DFT calculations [88]. Physisorption imposes difficulty in transferring charge due to the weak interaction of adsorbed CO_2_ with the surface. It was found that, approximately −0.06 electron charge transferred from the surface of metals listed in Table 3 (little or no dependence on the kinds of metals) as a result of the location of adsorbed CO_2_ (~3 Å) above the surface [88]. On the contrary, chemisorption states are highly dependent on both the metal itself and adsorption sites. Chemisorbed CO_2_ molecules often have a bent structure with O–C–O angle varying between 121 and 140° compared with the nearly linear coordination of the physisorbed CO_2_ molecules, and their extent depends on the kind of metal surface. In other words, the degree of CO_2_ activation varies with metal surfaces. A similar behavior can be observed for the amount of charge transferred (Table 3). According to Wang et al. [89], who investigated CO_2_ chemisorption on nine transition metal surfaces (Fe, Co, Ni, Cu, Rh, Pd, Ag, Pt, and Au), the adsorption strength is affected by both the *d*-band center of the metal surface and the charge transfer, which control the activation of C=O bond. It is possible to promote chemisorption by adjusting the properties of the catalysts, such as the catalyst surface area, surface defects, basic sites and the addition of promoters [31].

**Scheme 3:**
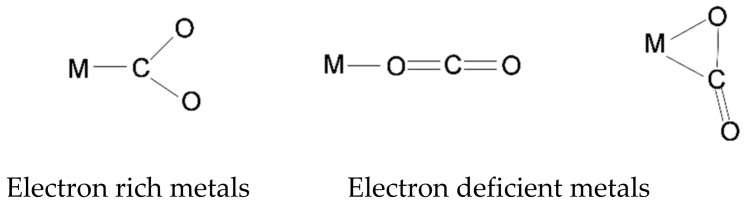
CO_2_ coordination modes at the transition metal center.

Generally, the DFT study of the CO_2_ activation on transition metal surfaces suggests different barriers for CO_2_ dissociation on different monometallic or bimetallic surfaces [88]. The coordination of CO_2_ with metals can take different modes, e.g., with the electron-deficient C atom as the electron acceptor and the C=O bonds or O atoms as the electron donor as shown in Scheme 3 [85]. Electron-rich metal surfaces such as Ni (110) and Cu (100) generally activates CO_2_ by attaching to the carbon atom [90,91]. In the process, electrons in the d_z_^2^ orbital of the metal transfer to the unpopulated antibonding π* orbital of the CO_2_ molecule. Consequently, negatively charged surfaces are efficient for CO_2_ activation via the charge transfer mode. Such charged surfaces can be generated through inserting strong metal-support interaction, bimetallic and ligand effects, etc. [85]. Electron-deficient metal surfaces, such as Ti, Cr, V, and Mn, favor the end-on coordination with CO_2_ [92]. In this mode, the bending or distortion of the linear CO_2_ molecule is difficult [85]. For metal centers with combined binding behavior, i.e., possessing both an electron acceptor site and an electron donor site, CO_2_ adsorption generally prefers the bridge sites. More efficient activation can result through this mechanism and lead to bending of the linear CO_2_ molecule from different sites [85].

Investigations by DFT calculations revealed the characteristic adsorption and activation of CO_2_ on Rh, Pd, Pt, Ni, Fe, Cu, Re, Al, Mg, and Ag metals [87,93]. Strong evidence has been provided for the formation of CO_2_^−^ according to spectroscopic results. Depending on the type of metal, CO_2_ can also dissociate into CO and O or be transformed into CO_3_^2−^ and CO. The activation of CO_2_ on Pt, Rh, Ni, Cu, Ag, and Pd followed different elementary steps as a result of the different levels of interaction of the metals with adsorbed oxygen in the RWGS reaction. Metals with high affinities toward oxygen presented lower activation barriers, leading to facile hydrogenation reactions [87]. The presence of preadsorbed oxygen was responsible for forming carbonates of different structures [87,93]. On most metal surfaces, CO_2_ activation is highly surface orientated, pressure- and particle size-dependent [89,94,95,96]. Yu et al. [96] demonstrated through spin-polarized DFT calculations that adsorption and dissociation of CO_2_ was dependent on the Co particle size. They showed that Co_55_ nanoclusters had the highest CO_2_ dissociation activity in comparison to Co_13_ and Co_38_. However, Co_13_ activated CO_2_ with the smallest O–C–O angle (123°) against 137° for both Co_38_ and Co_55_ nanoclusters.

**Figure 5:**
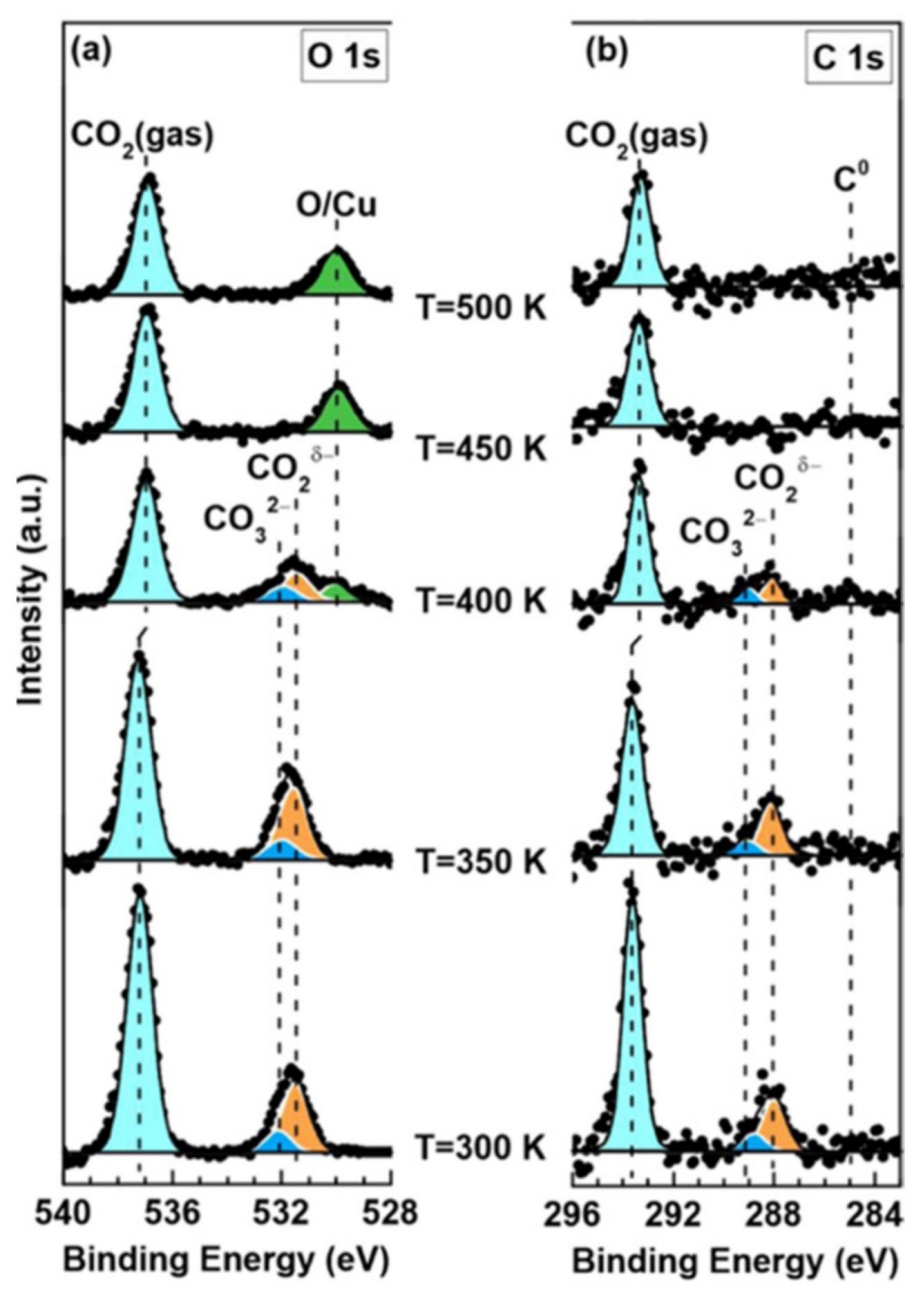
Ambient pressure XPS spectra of Cu (111) obtained in under 1 mbar CO_2_ at 300 K and at 350, 400, 450, and 500 K; (**a**) O 1s and (**b**) C 1s. Reproduced with permission from the authors of [95]. Copyright 2020 American Chemical Society.

Cu-based materials have gained much attention in the CO_2_ conversion process due to their wide applicability in the different conversion processes and low cost [12,97]. Despite these and other massive studies, the activation of CO_2_ on Cu catalysts is still an issue due to the poor understanding of its mechanism. Studies have shown that CO_2_ interacts weakly on low-index Cu surfaces under UHV conditions [98,99]. However, a recent study found Cu (100) surface to be more active in dissociating CO_2_ than Cu (111), producing oxygen [91]. Ambient pressure X-ray photoelectron spectroscopy (APXPS) and DFT calculations revealed the activation of CO_2_ on Cu surfaces. APXPS showed that CO_2_ adsorbed as CO_2_^δ^^−^ on Cu (111) surface under a pressure of 0.01 mbar at 300 K (Figure 5). With an increase in pressure to 1 mbar, adsorbed CO_2_^δ^^−^ partially transformed into carbonate as a result of the disproportionation reaction between CO_2_ molecules. Subsequent annealing at 400 K or higher temperatures led to the dissociation of CO_2_^δ^^−^ and carbonate and the formation of a chemisorbed oxygen-covered surface. On Cu (110) surface, the CO_2_^δ^^−^ gradually dissociated into CO and chemisorbed oxygen under the same CO_2_ pressure at room temperature. On both surfaces, atomic oxygen was generated that catalyzed the self-deactivation of CO_2_ adsorption. The DFT results, which collaborated the experimental findings, further indicated that the Cu (110) surface was more active than the Cu (111) surface in breaking C–O bonds [95]. Comparing the adsorption of CO_2_ on Cu (111), (100), and (110) surfaces, it was found that CO_2_ molecules aligned parallel to Cu (111) and (100), whereas a vertical configuration was more stable for the adsorbed CO_2_ on Cu (110) with one of two oxygen atoms towards the surface. The degree of CO_2_ activation followed the order: Cu (110) > Cu (100) > Cu (111) [89]. A decrease in the activation energies for CO_2_ dissociation has been observed when Cu surfaces have step or kink defects in comparison with the flat surface. However, chemisorption of CO_2_ has been reported on Cu stepped surfaces [98,99,100,101].

**Figure 6:**
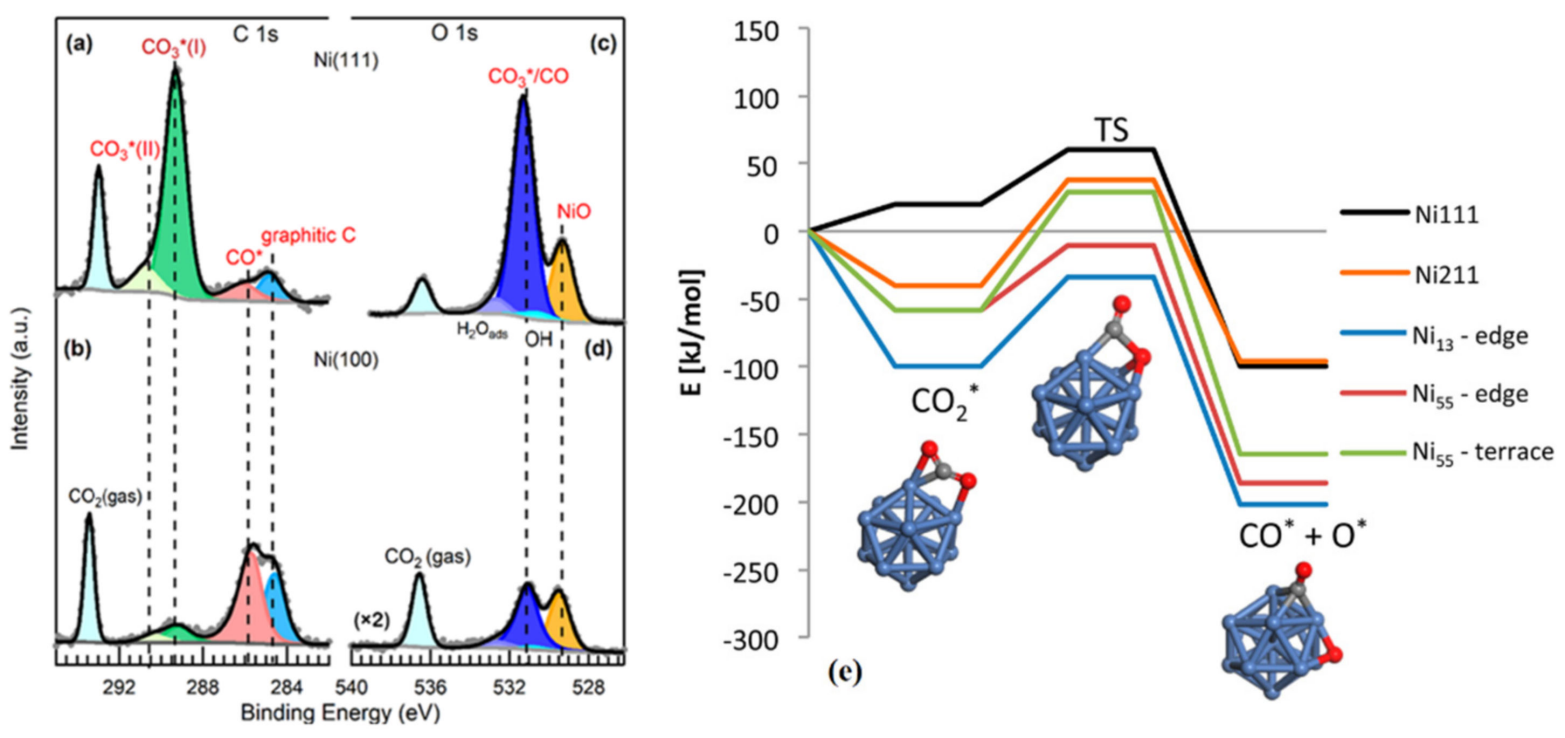
Ambient pressure XPS spectra of (**a**,**b**) C 1s, and (**c**,**d**) O 1s Ni (111) and Ni (100), respectively. CO_2_ adsorption was performed under 0.2 Torr CO_2_ at room temperature. Reproduced with permission from [107]. Copyright 2019 American Chemical Society; (**e**) Energy profile diagram for the CO_2_ activation on Ni (111) and Ni (211) surfaces, edges of Ni_13_ particle, and edges and terraces of the Ni_55_ particle and the activation geometries on the Ni_13_. Reproduced with permission from [108]. Copyright 2016 American Chemical Society.

Ni-based catalysts can dissociate and convert CO_2_ into value-added products, such as methane; thus, a fundamental understanding of the interaction between CO_2_ and Ni surface at the atomic level is crucial to design even more efficient Ni-based catalysts. According to theoretical studies, the surface orientation of Ni influences the activation of CO_2_ by altering the energetics for subsequent C–O bond breakage [102]. Ab initio calculations using slab models have shown that CO_2_ reactions on model Ni are surface sensitive, with reactivity in the following trend: Ni (110) > Ni (100) > Ni (111) [102,103]. Experimental investigations revealed the capability of Ni (110) surface to molecularly adsorb and subsequently dissociate CO_2_ at room temperature [104]. By using in situ APXPS, carbonate was identified as the dominating surface intermediate at room temperature upon CO_2_ adsorption on Ni (111) and Ni (110) surfaces [105,106,107]. However, where there are multiple dissociation products, their distribution depends on the surfaces. Carbonate, CO, and graphitic carbon were all observed on both Ni (111) and Ni (100) surfaces under a CO_2_ pressure of 0.2 Torr. Ni (111) was predominantly covered with carbonate, whereas adsorbed CO* and graphitic carbon were prevalent on the Ni (100) surface as indicated in Figure 6a–d [107]. The CO_2_ adsorption and dissociation on ideal Ni (111) and stepped Ni (211) surfaces are shown in Figure 6e. It can be seen that CO_2_ adsorption is endothermic by 20 kJ·mol^−1^ on the Ni (111) surface, whereas it is exothermic by 40 kJ·mol^−1^ on the Ni (211) surface [108].

Based on the experimental results of the ultrahigh vacuum (UHV), Auger electron spectroscopy (AES), temperature-programmed desorption (TPD), and high-resolution electron energy loss spectroscopy (HREELS), it is difficult for CO_2_ to adsorb on a clean Pt (111) surface between 110 and 300 K, but the dissociation capability can be improved by doping alkali metals such as potassium [109,110]. Conversely, it was reported that Pt foil treated with CO_2_ in the gas phase during in situ UHVXPS experiments at 77 K formed chemisorbed CO species [111]. In collaboration with the latter assertion, on the clean Pt (111) surface, CO_2_ dissociated into adsorbed CO and O at both room and elevated temperatures [112]. The production of adsorbed CO increased upon the introduction of H_2_ (hydrogenation reaction). This observation was obvious for the CO_2_ adsorption at all temperatures and led to the deoxygenation (consumption of oxygen) of the surface, resulting in cleaning the sites for further CO production and desorption from the surface at elevated temperatures. The Pt surface was active in the RWGS reaction. At a low pressure, the RWGS was initiated at 300 °C; on the contrary, at a high pressure (H_2_:CO_2_ of 150 mtorr: 15 mtorr), a low temperature (200 °C) favored the initiation of the RWGS reaction, and the conversion of CO_2_ continued to increase with increasing temperatures [112]. Moreover, under a pressure of 40 mtorr of pure CO_2_ and at temperatures below 150 °C, graphitic carbon has been also observed as a product of the Boudouard reaction. IR spectroscopy revealed the size-dependent nature of CO_2_ adsorption on Pt clusters. On small Pt clusters anions (Pt_n_^−^, n = 4–7), CO_2_ was highly activated but remained molecularly adsorbed on Pt_4_^−^. On large clusters, dissociative adsorption was observed [13].

The CO_2_^δ−^ state of adsorbed CO_2_ molecule has been observed on Co (100) and Co (110) surfaces upon CO_2_ dissociation based on the energetics, geometries, vibrational frequencies, and charge transfer analysis [113].

### 3.2. CO_2_ Activation on Bimetallic/Alloyed Catalyst Surfaces

Bimetallic surfaces are highly unique and active for a wide range of CO_2_ transformation reactions due to the electronic and geometric alterations within the structure. These alterations could be observed as a change in the morphology of metal, adsorption mode and configuration, and chemical ordering with varying composition and particle size [8,34]. This can help to control the adsorption properties to attain desired adsorption coverages. Numerous studies have appeared in the open literature discussing both activation of CO_2_ and the subsequent conversion of CO_2_ to fuels and chemicals on bimetallic catalysts. Ko et al. [88] studied the CO_2_ activation and adsorption by performing DFT calculations on a range of bimetallic alloy surfaces. The dissociation energy barriers of CO_2_ were screened by combining Bronsted–Evans–Polanyi (BEP) relation, scaling relation, and surface mixing rule. It was found that CO_2_ dissociated into CO and O, which sum of their adsorption energies was linearly related to both the energy for CO_2_ dissociation and that for CO_2_^δ−^ adsorption. The activation of CO_2_ proceeded through a direct dissociation (CO_2_ → CO + O) mechanism in three successive elementary steps: physisorption of CO_2_ from the gas phase on the metal surface, chemisorption of CO_2_^δ−^ from the physisorbed CO_2_, and direct dissociation of CO_2_^δ−^ into CO and O.

**Figure 7:**
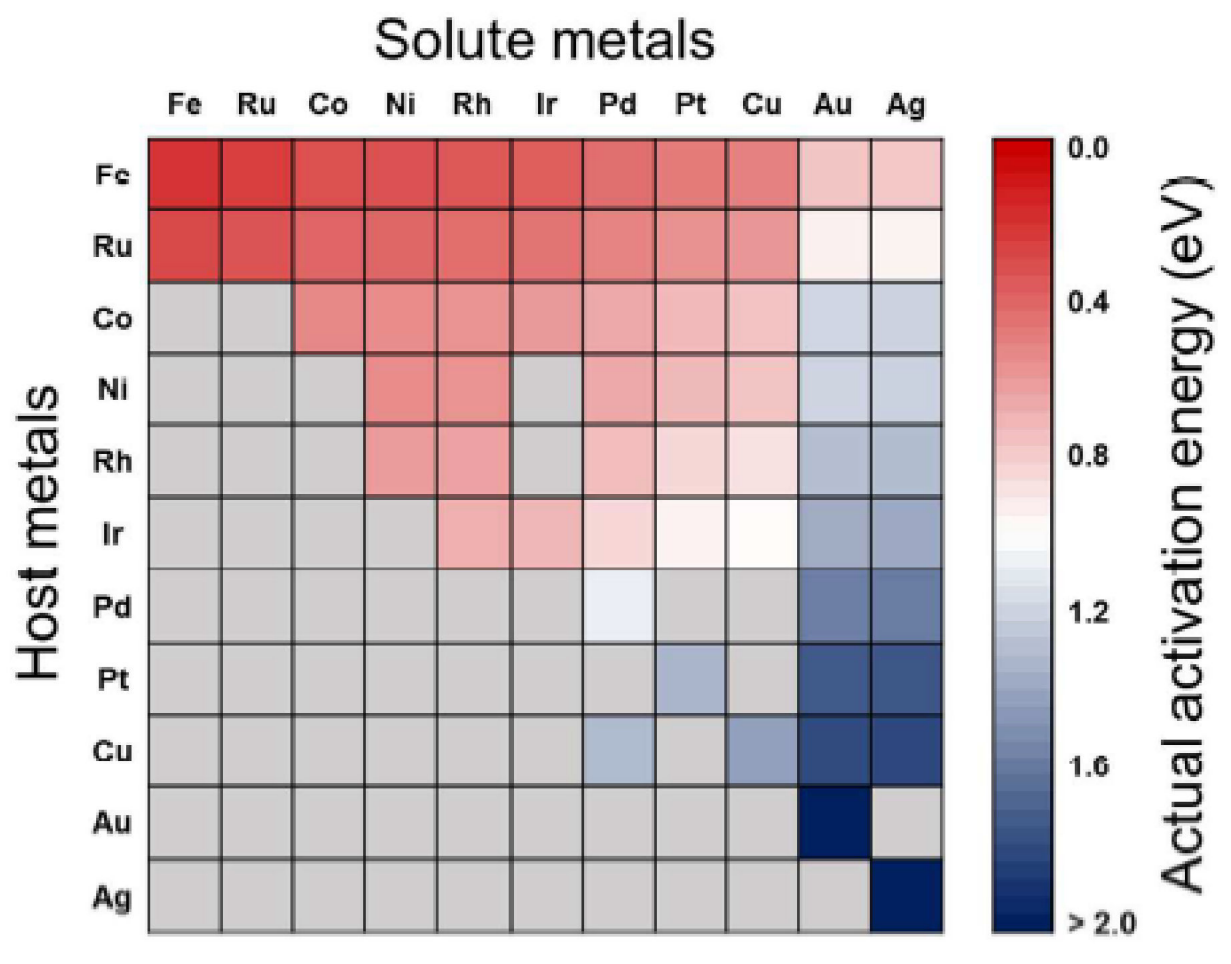
Screening for *E*_a_^act^ for CO_2_ dissociation on pure metals and bimetallic alloys. Gray cells indicate the bimetallic alloys which are not preferred to the surface segregation of solute atoms. *E*_a_^act^ were estimated by combining BEP relation, scaling relation, and surface mixing rule. Reproduced with permission from the authors of [88]. Copyright 2016 American Chemical Society.

The predicted activation energies for the CO_2_ dissociation on bimetallic alloy surfaces are shown in Figure 7, with the row and column indicating the host and solute metals of the bimetallic alloys, respectively. The alloying effect would lead to reduction for surface reactions when the solute metals are placed in the bulk region. In the figure, the activation energy (*E*_a_^act^) decreases from right to left and from bottom to top, thus alloys with relatively low activation energies (~0.75 eV) are Fe-, Ru-, Co-, Ni-, Rh-, and Ir-based alloys, whereas Pd-, Pt-, and Cu-based alloys possess high activation energies (~1.51 eV). Still, some metals, including Ru-, Co-, Ni-, Rh-, Ir-, and Cu-based alloys, have activation energies in between the extremes (0.76–1.50 eV). This picture makes sense for fabricating bimetallic catalysts for CO_2_ conversion reactions by manipulating the activation energies [88].

The mechanisms of CO_2_ dissociation on bimetallic clusters (Pt_3_Ni_1_, Pt_2_Ni_2_, and Pt_1_Ni_3_) were investigated and characterized by the activation barrier [21]. Compared with the single metallic clusters (Pt_4_ and Ni_4_), results showed the activation barrier to be between those of the monometallic clusters. Increasing Ni atoms in the bimetallic clusters moderately raised the activation barrier, which means that such a bimetal combination could have a significant negative impact on the activity of Pt for CO_2_ reaction. In the presence of H_2_, CO_2_ dissociation became less difficult for all metallic clusters [21].

Results from an ab initio study of chemisorption and activation of CO_2_ on Pt-based transition-metal nanoalloys on 55-atom nanoclusters (Pt_n_TM_55−n_), where n = 0, 13, 42, 55, and TM = Fe, Co, Ni, Cu, Ru, Rh, Pd, Ag, Os, Ir, Au, indicated a linear correlation between the interaction energy, charge transfer from the nanoclusters toward CO_2_ and the bent CO_2_ angle [8]. It was further realized that the interaction energy was enhanced for larger angles and molecular charge. With 55 atoms for Cu, Ag and Au in the Pt alloy, a change from physisorption to chemisorption was observed, whereas the strong interaction energy of CO_2_ with Os_55_, Ru_55_, and Fe_55_ can be decreased by alloying with Pt [8]. It was concluded that certain metals (Fe, Co and Ni) activate CO_2_ more strongly as monometals than in an alloyed form due to weaker adsorption energy in the latter.

### 3.3. CO_2_ Adsorption and Activation on Metal Oxide Surfaces

#### 3.3.1. Metal Oxide

**Scheme 4:**
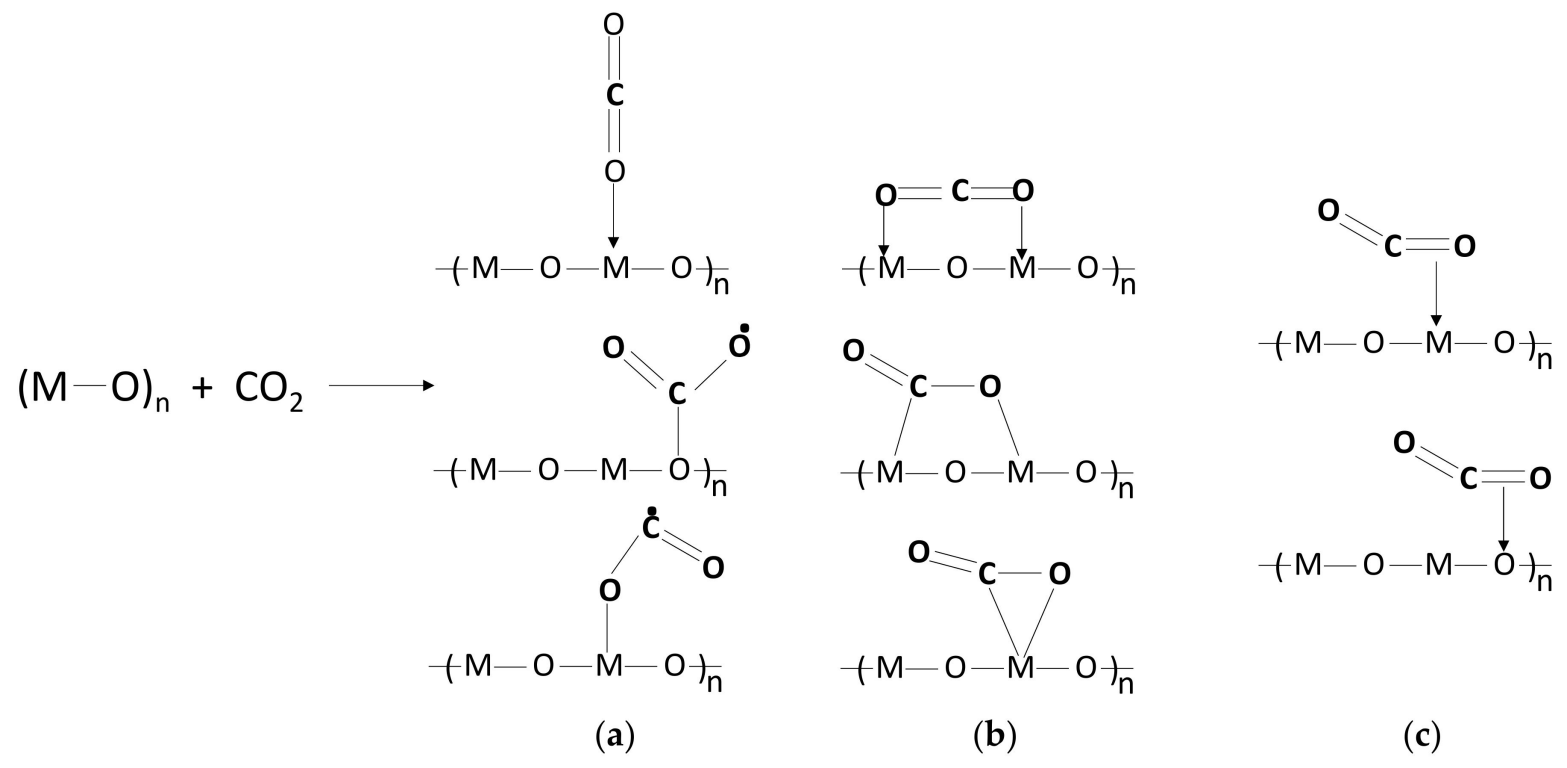
Possible modes of CO_2_ adsorption on metal oxide surfaces: (**a**) monodentate, (**b**) bidentate (**c**) π-coordination. Reproduced with permission from the authors of [114]. Copyright 2017 The Royal Society of Chemistry.

Various metal oxides (MO_x_) or (M_x_O_y_) are investigated as supports or directly as catalysts for CO_2_ conversion, including In_2_O_3_, CeO_2_, ZnO, ZrO_2_, TiO_2_, and CeO_2_. Metal oxide surfaces consist of both metal (M^n+^) and oxygen (O^2−^) ions, which are effective sites for CO_2_ activation. The activation can occur by coordination to one or two adjacent metal sites through the terminal oxygen atoms or C atom of the CO_2_ molecule, forming monodentate or bidentate carbonate species, as illustrated in Scheme 4a,b [114]. Interaction of the C atom is on the surface oxygen sites of the metal oxide. The CO_2_ activation can also occur via the σ-bond and π-bonds activation on metal ions and oxygen ions, respectively, as observed upon chemisorption on metal oxides applied as catalyst supports (as shown in Scheme 4c) [115]. Due to the large surface areas, switchable redox properties, and rich oxygen vacancies, metal oxides can act as adsorption and activation sites for small molecules, including O_2_, H_2_, and CO_2_ [116]. The surface oxygen vacancies interact with the carbon and/or oxygen atoms of CO_2_ through which electron transfer from the oxide defective site to adsorbed CO_2_ becomes feasible. One example is In_2_O_3_, which is rich in oxygen vacancies and has shown a high activity for CO_2_ activation and methanol synthesis by hydrogenation [116]. On oxide surfaces, generally, CO_2_ interactions can vary from physisorption to chemisorption, the extent that affects the structure and reactivity of the adsorbed CO_2_ and the kinetics and mechanisms of surface catalytic reactions [114]. The surface structure is important for CO_2_ adsorption and activation [117]; thus, the interaction of CO_2_ with metal oxides can be structure-dependent. It was found that CO_2_ adsorption on Zn_2_GeO_4_ (001) was higher than that on Zn_2_GeO_4_ (010) surface [117]. The interaction with (010) surface led to bidentate carbonate species, whereas on the (001) surface, stronger interaction with CO_2_ resulted in a bridged carbonate-like species. The strongest adsorption based on calculated CO_2_ adsorption energies were around the surface oxygen vacancy site on both surfaces. Analysis of the LDOS and Mulliken charge for adsorbed CO_2_ on perfect Zn_2_GeO_4_ surfaces revealed that CO_2_ formed a CO_2_^δ−^ species upon receiving electrons from the surface. CO_2_ molecule was found to be activated on the CuO surfaces ((011), (111), and (−111)), with strong adsorption only on the (011) surface. The CuO (111) and CuO (−111) surfaces showed relatively weak adsorption. CO_2_ activation was characterized by structural transformations and charge transfer that resulted in the formation of bent CO_2_^δ−^ species with an elongation of the C–O bonds [94].

The ability of metal oxides to bind and activate CO_2_ depends greatly on several factors, including their preparation methods, physiochemical properties, redox properties, and electronic and geometric structures [108,114,118]. The preparation methods were found to impact the properties of CeO_2_ nanostructures for the photocatalytic reduction of CO_2_ [119]. A high surface area was obtained for catalysts synthesized through the sunlight-assisted combustion process, in addition to possessing a small particle size, high concentration of oxygen vacancies, and a narrow bandgap. Compared to that prepared from the conventional combustion process with a spongy-like structure, a porous network consisting of small and uniformed pores was also obtained for the sunlight-assisted process. The superior catalytic properties could be attributed to the combustion process aided by solar irradiation during the synthesis process. As demonstrated with the CeO_x_/Cu catalyst, the important roles of metal oxide ions were revealed on the catalytic cycle of H_2_O and CO_2_ activation. The Cu phase reduced into Cu^0^ that promoted Ce^4+^ reduction into Ce^3+^. H_2_O and CO_2_ activation occurred on the Ce^3+^ sites. Without the presence of Cu, Ce^3+^ would lead to oxidation into Ce^4+^. However, in contact with Ce^4+^, Cu^0^ reacted to form Cu^+^ and Ce^3+^, sustaining ceria in the more active state. The cycle is closed when Cu^+^ reduced to Cu^0^ [120]. This synergistic effect afforded the catalysts with high reactivity in the RWGS reactions. The chemisorption of CO_2_ molecules on CeO_2_ at RT as studied using in situ DRIFTS indicated adsorption at both the Ce^3+^ and Ce^4+^ sites, although adsorption was also found at the oxygen sites that resulted in carbonates and bicarbonates species [121]. In the same study, the CO_2_ chemisorption on TiO_2_ under similar reaction conditions and instrumentation was observed at both Ti^3+^ and Ti^4+^ sites, exhibiting O–C–O vibrations at 1667 and 1248 cm^−1^ and 2339–2345 cm^−1^, respectively [121]. The CO_2_ molecules adsorbed at Ti^3+^ sites formed CO_2_^−^ species, which concentration increased with the amount of oxygen vacancies present. Like with CeO_2_, CO_2_ chemisorption at the oxygen sites formed carbonates and bicarbonates species. It was observed that the interaction between TiO_2_ and CO_2_ molecules is somewhat weak compared with that of CeO_2_. Such weak interactions can be improved by doping TiO_2_ with CeO_2_. CeO_2_ doping can improve the interaction of TiO_2_ with CO_2_ as a result of the introduction of Ce^3+^, which strengthens the bonding of CO_2_ with catalyst surfaces and enhances the production of bidentate carbon species that can readily be transformed to surface CO_2_^−^ in the presence of H_2_O under sunlight irradiation. The formation of adsorbed species of CO_2_ over CeO_2_/TiO_2_ could be attributed to the binding of CO_2_ species to Ti/Ce atoms that have reductive capabilities under photo-irradiation. Furthermore, the Ce^3+^ availability from CeO_2_ facilitates photogenerated charge separation; thus, the CO_2_ adsorption and enhanced charge separation can be tuned for increased activity of CeO_2_/TiO_2_ catalyst [122]. The surface area of a material positively correlates with its adsorption capacity. It was found that the Bi_12_O_17_Cl_2_ nanotubes had higher adsorption capacity for CO_2_ (~4.3 times) than bulk Bi_12_O_17_Cl_2_ due to the higher BET specific surface area of the former. As a result, the effective adsorption of CO_2_ on Bi_12_O_17_Cl_2_ nanotubes than bulk Bi_12_O_17_Cl_2_ favored the photocatalytic process [123]. In addition, the high surface area correlated with strong adsorption. Weak chemisorption of CO_2_ has been reported for CeO_2_ nanostructures with low exposed surface area [121]. Mesoporous structured photocatalyst displayed improved activity for CO_2_ reduction into CH_4_ due to the presence of high specific surface area and mesostructure that enhanced adsorption of CO_2_ [124]. Highly mesoporous In(OH)_3_ synthesized via a sol–gel hydrothermal treatment exhibited ~20-fold higher efficiency for CO_2_ reduction in comparison with that lacking mesopores [125]. It is reported that the methanol activity of the In_2_O_3_ catalyst could also be improved by increasing the (111) surface area [126].

#### 3.3.2. Characteristic Adsorption of Representative Metal Oxides

Ceria (CeO_2_) has shown catalytic activity in the reduction of CO_2_ to liquid fuels and chemicals. It has rich oxygen vacancies and a high oxygen storage/release capacity. Several studies demonstrating the interaction of CO_2_ with high-surface-area ceria catalysts have been reported. As noted in [127], CO_2_ dissociates into CO and an oxygen-containing surface species on the surface Ce^3+^ ions, which are considered active sites for CO_2_ activation due to formation of carbonates or inorganic carboxylates. Graciani et al. reported a highly active CeO_x_/Cu nanoparticles catalyst for methanol synthesis from CO_2_ [128]. The catalyst activated CO_2_ as CO_2_^δ^^−^ and exhibited a faster methanol production rate than Cu/ZnO, on which CO_2_ was chemisorbed as CO_3_^2^^−^. A study on the CO_2_ adsorption sites of CeO_2_ (110) surface using DFT was carried out by Cheng et al. [129]. Reduced and stoichiometric ceria (110) surfaces were compared. Results revealed that CO_2_ adsorption on the reduced ceria (110) surface was thermodynamically favored than on the stoichiometric ceria (110) surface. Furthermore, the most stable adsorption configuration consisted of CO_2_ adsorbed parallel to the reduced ceria (110) surface at the oxygen vacancy. Upon adsorption, the CO_2_ molecule distorted out of the plane and formed carbonates with the remaining oxygen anion at the surface [129]. It was suggested that the structural changes in the catalyst after CO_2_ adsorption were due to charge transfer between the surface and adsorbate molecule. The formation of two different adsorbate species: a carbonate and a weakly bound and linear physisorbed species, were observed upon exposure of reduced CeO_2−x_ (110) substrates to CO_2_ at low temperatures. There was no evidence for the formation of CO_2_^δ^^−^. Furthermore, based on angle-dependent C K-edge NEXAFS spectra, the most preferred orientation of the adsorbate was lacking. The physisorbed CO_2_ species and carbonate species were completely desorbed at 250 and 500 K, respectively. The authors remarked that it is most unlikely that the activation of CO_2_ on the reduced CeO_2−x_ (110) surface was via breaking the C=O bond to form CO and O. However, on fully oxidized CeO_2_ (110), CO_2_ adsorbed as a carbonate which was completely decomposed and desorbed as CO_2_ at 400 K [130]. CO_2_ adsorbed on the CeO_2_ (111) surface formed a monodentate carbonate species found to be most stable on CeO_2_ at low coverages [131]. Increasing the CO_2_ coverage destabilized the formed species, indicating a mixed adsorption mechanism with both carbonate and linear adsorbed CO_2_ species. Although CeO_2_ has been studied for CO_2_ reduction reactions, the insights into CO_2_ adsorption, activation, and reaction on ceria surfaces are not yet fully understood.

TiO_2_ possesses good photocatalytic properties for many chemical reactions, including CO_2_ reduction. Since its first demonstration in the photoelectrochemical CO_2_ reduction to formic acid and formaldehyde by Inoue et al. [132], TiO_2_-based materials have attracted great research interests in CO_2_ photoreduction reactions. The adsorption properties of CO_2_ on both the rutile and anatase phases of TiO_2_ have been widely studied using various surface science techniques [133,134,135,136]. Sorescu et al. [137] investigated the adsorption and dissociation of CO_2_ on an oxidized anatase (101) surface using dispersion-corrected DFT and found CO_2_ to adsorbed at a fivefold coordinated Ti site in a tilted configuration. Based on in situ FTIR experiments, the CO_2_ adsorption formed CO_3_^2^^−^ and CO_2_ bonded to Ti, with absorption bands at 1319, 1376, 1462, 1532, 1579, and 2361 cm^−1^. The band at 2361 cm^−1^ was assigned to adsorbed CO_2_ with Ti–O–C–O adsorption configuration [138]. The 1319 and 1579 cm^−1^ bands were assigned to bidentate carbonate, while the band at 1461 cm^−1^ was due to monodentate or free carbonate. Under the vacuum condition, the intensities of all of the bands were reduced at 35 °C. The bidentate carbonate was the predominant species for CO_2_ on TiO_2_. The scanning tunneling microscopy (STM) enabled the study of the dissociation of CO_2_ adsorbed at the oxygen vacancy of TiO_2_ (110) at the single-molecule level [139]. It was found that the electrons injected from the STM tip into the adsorbed CO_2_ caused its dissociation into CO and O, and the released O was observed to heal the oxygen vacancy. According to experimental analysis, ~1.4 eV above the conduction band minimum of TiO_2_ is needed for the electron induction process to dissociate CO_2_. The formation of a transient negative ion by the injected electron is an important step in the CO_2_ dissociation, and this can only be possible above the threshold voltage. TiO_2_ modified with metal oxide nanoclusters possess enhanced activity to adsorb and convert CO_2_ [26,140]. The Bi_2_O_3_-TiO_2_ heterostructures obtained by modifying TiO_2_ with Bi exhibited low coordinated Bi sites in the nanoclusters and a valence band edge consisting mainly of Bi–O states due to the presence of the Bi lone pair. Upon interaction of CO_2_ with the reduced heterostructures, CO or CO_2_^−^ were observed mainly through electron transfer to CO_2_, and the Bi_2_O_3_–TiO_2_ heterostructures became oxidized in the process with adsorbed CO_2_ in carbonate form [140]. In a related study, clean or hydroxylated extended rutile and anatase TiO_2_ surfaces modified with Cr nanoclusters presented an upshift valence band edge related to the existence of Cr 3d–O 2p interactions, which promoted the CO_2_ activation. [26]. The activated CO_2_ molecule reduced its O–C–O angle to 127–132° and increased the C–O bond length 1.30 Å. It was concluded that the strong CO_2_–Cr–O interaction induced the structural distortions.

Iron oxides (FeO_x_) are an important component of catalysts for the conversion of CO_2_ to hydrocarbons (liquid fuels). The adsorption and activation of CO_2_ on FeO_x_ have been investigated by researchers [141,142,143]. It is suggested that Fe^2+^ and Fe^3+^ cations are crucial for CO_2_ adsorption. Using TPD, Pavelec et al. [141] observed a weak interaction between CO_2_ and Fe_3_O_4_ (001) surface. On this surface, CO_2_ molecules existed in the physisorbed state as they desorbed at a low temperature (115 K). However, strong CO_2_ adsorption was observed on the defects and surface Fe^3+^ sites. Weak CO_2_ adsorption has also been observed on Fe_3_O_4_ (111) as investigated by various experimental techniques [144]. At different CO_2_ dosages and temperatures (between 120 and 140 K), TPD experiments suggested CO_2_ adsorb very weakly on a regular Fe_3_O_4_ (111) surface. However, CO_2_ chemisorption was also observed but at relatively long CO_2_ exposure times [144,145] via binding to under-coordinated oxygen sites [145]. The formation of chemisorbed species such as carboxylates and carbonates was facilitated by surface imperfections. Conclusively, FeO_x_ exhibit weak interaction with CO_2_ molecules, and studies are recommended in this direction to adjust its CO_2_ adsorption strength.

ZrO_2_ has been demonstrated as an active catalyst or catalyst support for the CO_2_ hydrogenation reactions to a variety of products. In a study on CO_2_ hydrogenation on Cu/ZrO_2_ interface using the first-principles kinetic Monte Carlo simulations by Tang et al. [146], the authors showed that CO_2_ prefers to adsorb on the bare ZrO_2_ nanoparticles surface rather than at the Cu/ZrO_2_ interface. This led to the bending of the CO_2_ molecule with a calculated adsorption energy of 0.69 eV. The stretching of the C–O bonds and charge transfer from the ZrO_2_ surface to the antibonding 2π_μ_ orbital of CO_2_ were also observed. On the bare ZrO_2_ surface, bidentate bicarbonate (HCO_3_) was formed upon CO_2_ adsorption based on observable IR frequencies at ~1225, ~1620, and ~3615 cm^−1^ [147].

#### 3.3.3. Oxygen Vacancies and Their Roles in CO_2_ Activation

Many metal oxides, particularly the reducible ones (e.g., TiO_2_, CeO_2_, and In_2_O_3_), contain certain number oxygen vacancies, depending on the preparation and treatment conditions. The formation of oxygen vacancies can alter the physicochemical and electronic properties, improve surface adsorption, and generate additional active centers of metal oxides [148]. As reported in the literature, the generation of oxygen vacancies in catalytic materials is linked with enhanced light harvesting, charge separation and transfer in photochemical reactions, charge (electron) production and transfer in thermochemical reactions, and charge density increase in electrochemical reactions. During CO_2_ conversion by the mentioned approaches, surface oxygen defects on metal oxides can significantly influence the interaction of CO_2_ with the surface and enhance the adsorption compared to the perfect surface due to modification of adsorption and binding properties [117]. In theory, removing O atoms in metal oxides leads to the formation of oxygen defects/voids that can activate CO_2_ due to the unsaturated chemical bond in the metal oxide and the unshared pair electrons of CO_2_ [72]. This reconfigures the coordination environment and delocalizes charge, facilitating the conversion of reaction intermediates [149]. The backward transfer of electrons from the defect to the adsorbed molecule alters the d-band center of the oxide catalyst, causing further interaction with the adsorbed CO_2_ and resulting in the reduction of activation energy barrier and enhanced CO_2_ reduction [72]. In summary, oxygen vacancies have valuable input to the overall reaction mechanism, thermodynamics, and kinetics.

##### How to Generate Oxygen Vacancies

Theoretical studies predict that reducible metal oxides with oxygen vacancies possess high CO_2_ adsorption and activation properties and can improve selectivity toward a characteristic reaction product [58,73,74,139,150,151,152]. Thus, the presence of oxygen vacancies in proper concentration can improve the catalytic performance of oxide-based catalysts. While oxygen vacancies in a catalyst sample can be detected by various techniques [153], X-ray photoelectron spectroscopy (XPS) is a good technique for confirming and measuring the relative oxygen vacancy concentration [119,154,155]. The ratio of the peak areas of lattice oxygen (O_lat_) to adsorbed oxygen (O_ads_), i.e., O_lat_/O_ads_ area ratio, obtained from the XPS can be used to estimate the relative concentrations of oxygen vacancy. A low oxygen vacancy concentration would exhibit a high O_lat_/O_ads_ ratio [154,155]. Oxygen vacancies can be formed during catalytic reactions [85] or be deliberately introduced in metal oxides during synthesis [153].

The formation of oxygen vacancies during reactions can be enabled by reaction conditions, via thermal desorption of lattice oxygen or reduction by reactive molecules (H_2_ or CO) [152,156,157,158]. Their generation through the former process is highly endothermic, while it is exothermic when initiated in the presence of H_2_ or CO; it is more exothermic using CO than H_2_ [152]. As an example, the surface reduction of In_2_O_3_ with H_2_ at above 500 K reordered the surface and formed defective sites [157]. During CO_2_ hydrogenation on oxide supported metal catalysts, H_2_ that dissociates on the metal site spills over and attacks the oxide at the metal-oxide interface, generating oxygen vacancies [156]. It was revealed in CO_2_ methanation over Ru/CeO_2_ catalyst, using operando XAENS, IR, and Raman that Ru species reduced to metallic Ru, which provided the ability to dissociate H_2_. The dissociated H atom attacked the Ce–O bond on the CeO_2_ surface, generating surface OH, Ce^3+^, and oxygen vacancies [156]. The hydrogen spillover effect of metallic Ru would facilitate the attacking of Ce–O bond by H atoms. Pd/In_2_O_3_ catalyst with highly dispersed Pd NPs (~3.6 nm in size) and predominately exposed (111) facets showed a better ability to dissociatively adsorb hydrogen and supply it for creating oxygen vacancy. The interfacial sites were also active for enhanced CO_2_ adsorption and hydrogenation [158].

Catalytic activities of irreducible metal oxides such as Al_2_O_3_, ZnO, and MgO can be improved by introducing oxygen vacancies in them. Methods including doping, plasma-assisted techniques, photo-irradiation of synthesis reaction, high-temperature annealing under inert environment, and wet and solid-state chemical reactions have been reported to create oxygen vacancies in metal oxides [74,119,153]. Doping suitable elements, both metallic (Cu, Fe, La, etc.) and non-metallic (C, N, S, P, etc.), is a proven method for introducing oxygen vacancies in metal oxides, which can be carried out either during synthesis or by post-synthesis treatment. For example, doping of non-metallic elements in TiO_2_ has been reported as an effective approach to producing oxygen vacancies [153]. Wang et al. [159] increased the oxygen vacancy concentration in CeO_2−x_ by doping Cu to it. Cu doping stabilized the pre-existed vacancies in CeO_2−x_ and resulted in improved and sustained photocatalytic activity in CO_2_ reduction. Co-doping or self-doping has also been reported. In co-doping, any two or more combinations of metals, metal/non-metal or non-metal/non-metal, can effectively dope and create oxygen vacancies [160,161]. Self-doping, on the other hand, occurs intrinsically within the metal oxide structure. Such structural doping can be achieved by other oxygen vacancies creation methods such as treatment with plasma, H_2_ or via redox reactions [162].

Solid-state and wet chemical redox reactions can produce metal oxides with sufficient oxygen vacancies, applying various treatments or reactions [153,163]. In the solid-state process, the reaction uses gaseous or solid reductants such as graphene, H_2_, NH_3_, S, CaH_2_, NaH, and LiH at high temperatures. The wet reaction is carried out in the liquid phase in the presence of a suitable reducing agent such as NaBH_4_ at room temperature or by using the hydrothermal process. The mechanism involves decreasing the oxidation states of the metal cations during the redox reactions, leading to the formation of oxygen vacancies. In addition, oxygen vacancies can be created in metal oxides by decreasing the particle size of the bulk material [56,164]. Guo et al. [164] introduced oxygen vacancies in Bi_2_Sn_2_O_7_ nanoparticles by decreasing the nanoparticle size to ~4 nm via the solution chemistry synthesis technique. The formation of oxygen vacancies was confirmed by an obvious increase in the visible light absorption according to the UV–visible spectroscopy results. The obtained oxidic nanoparticles with abundant oxygen vacancies exhibited an 8-fold enhanced photocatalytic performance compared with the bulk Bi_2_Sn_2_O_7_ in CO production from CO_2_ in pure water. Layered double hydroxide (LDH) nanosheets were synthesized via the templated hydrothermal methods (inverse microemulsion technique or controlled hydrolysis) [56]. These methods decreased the thickness of the Zn-containing LDH nanosheets from 5 μm to 40 nm, with platelet thicknesses of ≈2.7 nm. A combination of several characterization results (EXAFS, positron annihilation spectrometry, ESR, and DFT calculations) concluded that oxygen vacancies were created with decreasing LDH thickness. The created oxygen vacancies formed coordinatively unsaturated Zn with the nanosheets represented as Zn^+^–V_o_ complexes that serve as trapping sites for the efficient adsorption of CO_2_ and H_2_O molecules, promoting photoinduced charge separation, thus significantly improving the catalytic activity for CO_2_ photoreduction. These illustrations demonstrate the possibility of engineering metal oxide catalysts with abundant oxygen vacancies for enhanced activity for CO_2_ reduction.

**Figure 8:**
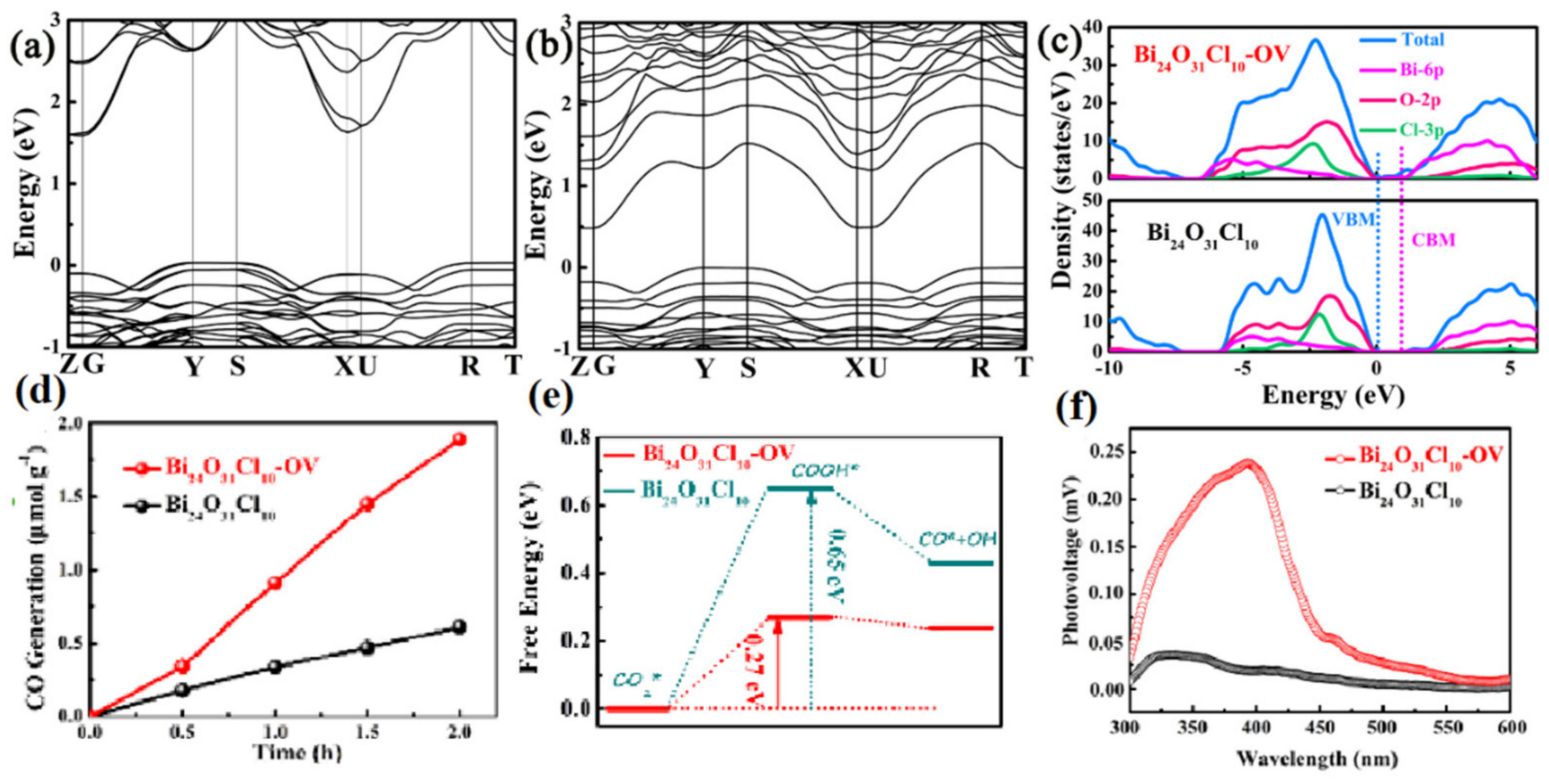
Calculated electronic band structures of (**a**) Bi_24_O_31_Cl_10_, (**b**) Bi_24_O_31_Cl_10_-OV, and (**c**) the corresponding DOS plots. (**d**) CO evolution rate of Bi_24_O_31_Cl_10_ and Bi_24_O_31_Cl_10_-OV. (**e**) Free-energy diagram for CO_2_ reduction to CO on Bi_24_O_31_Cl_10_-OV. (**f**) Surface photovoltage spectroscopy showing carrier separation behavior. Reproduced with permission from the authors of [148]. Copyright 2019 John Wiley & Sons, Inc.

Thermal treatment of metal oxides in reducing gases (H_2_, Ar or vacuum) at a high temperature is a proven strategy to generate or enrich oxygen vacancies in metal oxides. The process ejects some lattice oxygen from the crystal and effects a change in the bulk phase. This method is very easy and efficient because the oxygen vacancy concentration can be easily tuned by simply controlling either the annealing temperature or reducing gas flow rate during treatment [163,165]. Ye et al. [154] applied a zeolite imidazolate framework-8 (ZIF-8)-derived ZnO as the carrier and synthesized carbon-modified CuO/ZnO catalyst with high oxygen vacancy content for CO_2_ hydrogenation to methanol. They revealed that the pyrolysis temperature of ZIF-8 affected the surface oxygen defects of ZnO, and the CuO/ZnO catalyst (pyrolyzed at 400 °C) achieved the best CO_2_ conversion and methanol selectivity due to the presence of more oxygen vacancies and carbon modification. Oxygen vacancies were engineered in bulk Bi_24_O_31_Cl_10_ by thermal annealing at 250 °C under an inert atmosphere consisting of H_2_/Ar (10/90 *v*/*v*) [148]. In the process, H_2_ interacted with the lattice oxygen, forming oxygen vacancies, which created a new level near the conduction band minimum, enabling a fast charge transfer and high carrier density (Figure 8a,b). As shown in Figure 8e, the existence of oxygen vacancies led to a reduction in the energy barrier for the *COOH intermediates formation during CO_2_ photochemical reduction. The photocatalytic activity of Bi_24_O_31_Cl_10_ for photoreduction of CO_2_ to CO was enhanced. CO was generated at a rate of 0.9 mmol h^−1^ g^−1^, which is 4 times higher compared with that of bulk Bi_24_O_31_Cl_10_ [148]. A controlled thermal process was adopted to prepare different indium oxide InO_x_ nanoribbons (NRs) with tunable O-vacancy: P-InO_x_ NRs, O-InO_x_ NRs, and H-InO_x_ NRs. The H-InO_x_ NRs rich in O-vacancy showed enhanced performance for the electrocatalytic CO_2_ conversion to HCOOH with the selectivity up to 91.7% [72].

In the plasma method, the material is subjected to high-energy ion (such as Ar^+^, N_2_^+^, H_2_O^+^) bombardment [166]. Metal oxides bombardment with these high-energy ions generates oxygen vacancies on the surface. An advantage is that the plasma treatment is fast and can effectively etch the oxide to expose more surface sites and selectively remove oxygen from the surface to produce oxygen vacancies. Geng et al. [74] introduced oxygen vacancies in ZnO nanosheets via H_2_ plasma treatment for the electrochemical reduction of CO_2_. This induced the formation of a new defect level around the valence band maximum, where abundant localized electrons accumulation caused increasing charge density. The new electronic structure promoted the activation of CO_2_, and the ZnO nanosheets with rich oxygen vacant sites (V_o_-rich ZnO nanosheets) exhibited a current density for CO production of −16.1 mA cm^−2^ with a Faradaic efficiency of 83% at −1.1 V versus RHE. The oxygen vacancies concentration can be controlled by the plasma treatment time. 

**Figure 9:**
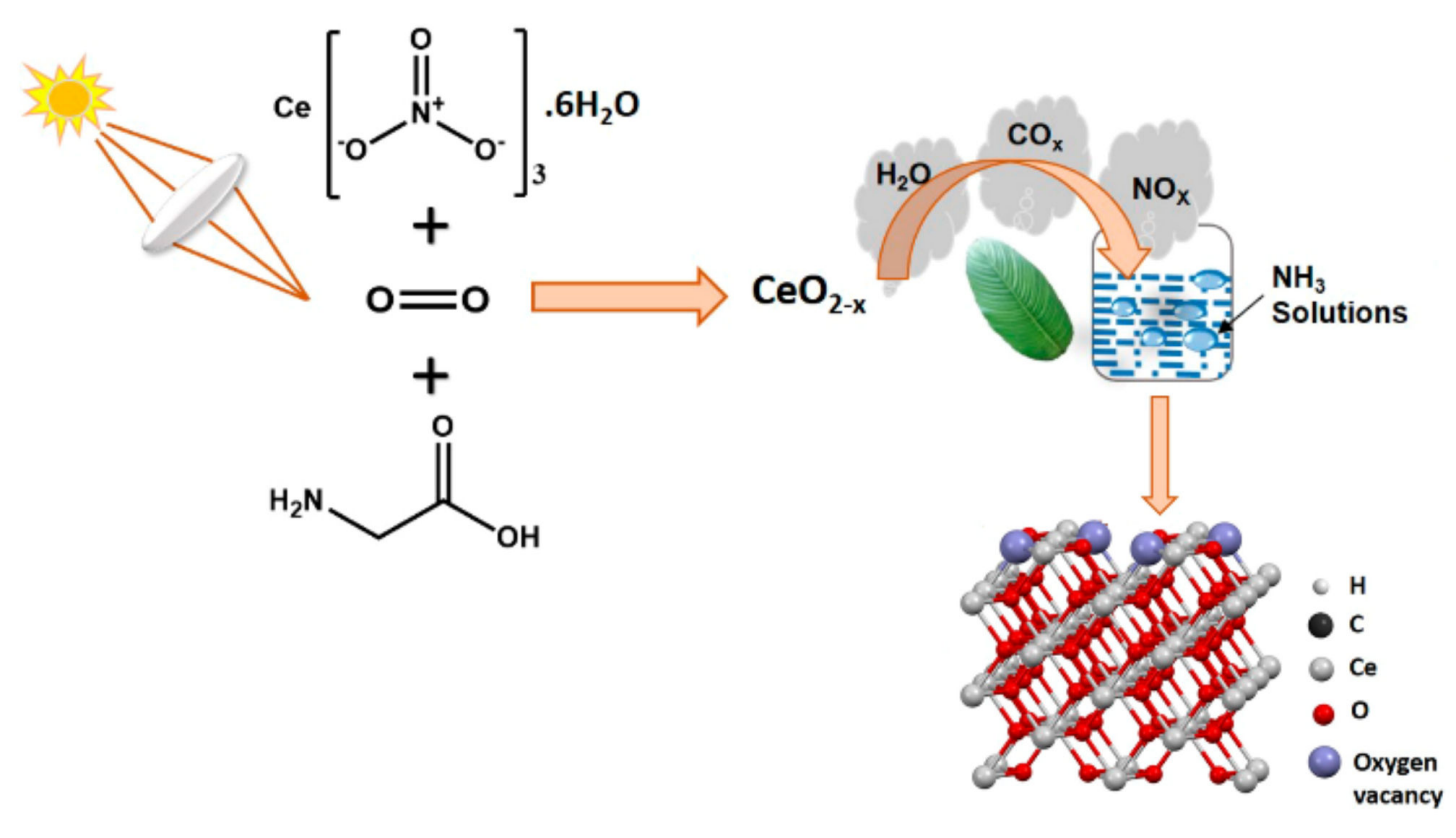
Photo-assisted solution combustion synthesis of CeO_2_ with rich oxygen vacancies. Redrawn with permission from the authors of [119]. Copyright 2019 American Chemical Society.

The photochemical reactions for generating oxygen vacancies involve directing a photo source to the synthesis reaction system, as shown in Figure 9 [119]. Hezam et al. [119] used sunlight as the energy source to effect exothermic combustion reaction between Ce-(NO_3_)_3_·6H_2_O and C_2_H_5_NO_2_. The presence of the light source improved the oxygen vacancies generation in CeO_2_ compared with CeO_2_ synthesized in the conventional way, without sunlight.

##### The Roles of Oxygen Vacancies

(1) CO_2_ adsorption and dissociation, creation of binding and active sites

In the adsorption and dissociation of CO_2_ on the anatase TiO_2_ (101) surface, by dispersion-corrected DFT calculations, Sorescu et al. [137] found that CO_2_ adsorbed at a fivefold coordinated Ti site in a skewed configuration on an oxidized surface. The presence of a bridging oxygen defect allowed for the creation of strong binding configurations. Lee et al. [139] found that CO_2_ was preferentially adsorbed at the oxygen vacancy defects on the TiO_2_ (110) surface. During the CO_2_ reduction process, one oxygen atom of CO_2_ filled an oxygen vacancy, decreasing the surface concentration. Oxygen vacancies provided active sites and increased the CO_2_ adsorption energy, promoting CO_2_ adsorption and activation on the photocatalyst surface. It was also found that subsurface oxygen vacancy enhanced the binding of CO_2_ molecules to the surface, and the CO_2_ dissociation from the defect sites was exothermic with a barrier of less than 21 kcal/mol [70]. CO_2_ adsorbed directly at the oxygen vacant sites of Zn_2_GeO_4_ dissociated into CO and O [117]. As the oxygen vacancies can be healed by oxygen atoms released during the dissociation process, the dissociative adsorption of CO_2_ on the oxide followed a stepwise mechanism that could be described as: CO_2_ + V_o_ → CO_2_^δ^^−^/V_o_ → CO_ad_ + O_sur_, where V_o_ is the oxygen vacancy.

**Figure 10:**
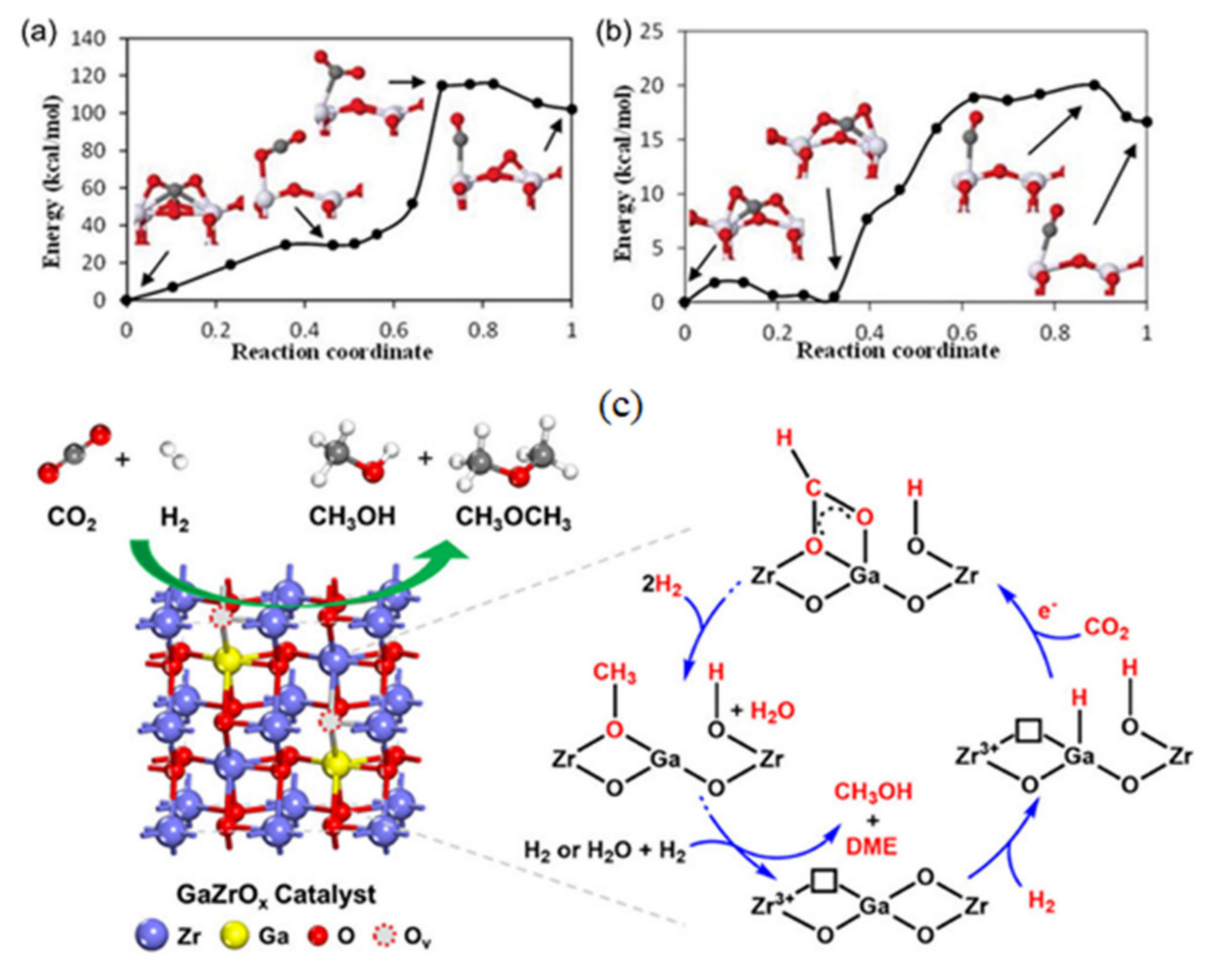
CO_2_ dissociation minimal energy pathway on (**a**) stoichiometric anatase (001) surface and (**b**) reduced anatase (001). Reproduced with permission from the authors of [150]. Copyright 2016 American Chemical Society (**c**) Proposed mechanism for the hydrogenation of CO_2_ to methanol and DME on the GaZrO_x_ catalyst. Reproduced with permission from the authors of [167]. Copyright 2021 American Chemical Society.

Huygh et al. [150] studied the adsorption and activation of CO_2_ on the fully oxidized and reduce anatase TiO_2_ (001) surfaces using DFT calculations with long-range dispersion energy corrections (Figure 10a,b). They found the monodentated carbonate-like structure to be the most stable adsorption configuration for the fully oxidized surface. CO_2_ dissociation was not observed on the stoichiometric anatase TiO_2_ (001) surface. The introduction of oxygen-vacant defects gave rise to new highly stable adsorption configurations with stronger C–O bond activation. These reactions caused the formation of a CO molecule, which will easily desorbed, and the reduced surface became oxidized.

By DFT slab calculations, Pan et al. [151] studied the effects of hydration and oxygen vacancy on CO_2_ adsorption on the β-Ga_2_O_3_ (100) surface. Adsorption of CO_2_ on the perfect dry β-Ga_2_O_3_ (100) surface was slightly endothermic, with an adsorption energy of 0.07 eV, which resulted in a carbonated species. The presence of oxygen vacancies promoted the adsorption and activation of CO_2_. On the most stable CO_2_ adsorption configuration, one of the oxygen atoms of the adsorbed CO_2_ occupied the oxygen vacancy site with low adsorption energy (−0.31 eV). Feng et al. [167] synthesized a series of GaZrO_x_ catalysts by the evaporation-induced self-assembly (EISA) method and proposed the Zr^3+^–O_V_–Ga–O species (O_V_ represents the oxygen vacancy) on the GaZrO_x_ surface as the active site (Figure 10c). The synergistic effect stems from neighboring Ga–O and Zr^3+^–O_V_ sites. H_2_ dissociated on the Ga–O sites to produce Ga–H and OH species, while CO_2_ was trapped by the oxygen vacancies and activated by electron transfer from the Zr^3+^ ions. Liu et al. [47] studied the activation of CO_2_ on the defective surface of Cu(I)/TiO_2−*x*_ using the in situ diffuse reflectance infrared Fourier transformed spectroscopy (DRIFTS). They observed the formation of CO_2_^−^ species upon the transfer of an electron to CO_2_. The anionic CO_2_^−^ species further dissociated into CO on a partially oxygen-depleted Cu(I)/TiO_2−*x*_ surface obtained by thermal annealing in a non-reactive atmosphere. The dissociation process was enhanced by the surface Cu^+^ species on the catalyst. The defective surface also delivered the electronic charge for the formation of Ti^3+^. It was observed that the dissociation of CO_2_^−^ in the absence of light related to the surface oxygen vacancies that provided electronic charge and sites for the adsorption of oxygen atoms from CO_2_. DFT studies also confirmed that the adsorption of CO_2_ at the oxygen vacancy site over the Zn-terminated ZnO (0001) surface occurs by inserting one of the O atoms of CO_2_ into the vacancy, resulting in the formation of a bent CO_2_ species [168]. Singh et al. [169] investigated the role of oxygen vacancies and basic site density in tuning methanol selectivity over Cu/CeO_2_, Cu/ZnO, and Cu/ZrO_2_ catalysts during CO_2_ hydrogenation. The strong basic sites for CO_2_ adsorption over catalyst surface were attributed to unsaturated, low coordination oxygen atoms (derived from oxygen vacancies), the oxygen vacancy concentrations of Cu/CeO_2_ catalyst was higher than those of Cu/ZnO and Cu/ZrO_2_ catalysts, so the Cu/CeO_2_ catalyst exhibited more than 90% methanol selectivity at 220 °C and 30 bar. The adsorption capacity of m-ZrO_2_ was increased due to the high concentration of oxygen vacancy on its surface, which led to the higher catalytic activity of Cu/m-ZrO_2_ compared with that of Cu/t-ZrO_2_ [170]. This benefited the activation of CO_2_, thus improving the catalytic activity for methanol synthesis. Martin et al. [171] synthesized In_2_O_3_/ZrO_2_ catalyst and observed the catalyst had high activity, high stability, and high selectivity due to the possession of a large number of oxygen vacancies. It was difficult to form oxygen vacancy on the Pt/SiO_2_ interface, and also weak CO_2_ adsorption was observed due to an obvious lack of the oxygen vacancies. However, CO_2_ adsorbed strongly on TiO_2_ due to the presence of oxygen vacancies on the TiO_2_ surface [172].

(2) Selection of reaction pathway, stabilization of key intermediates and electronic structure modification

**Figure 11:**
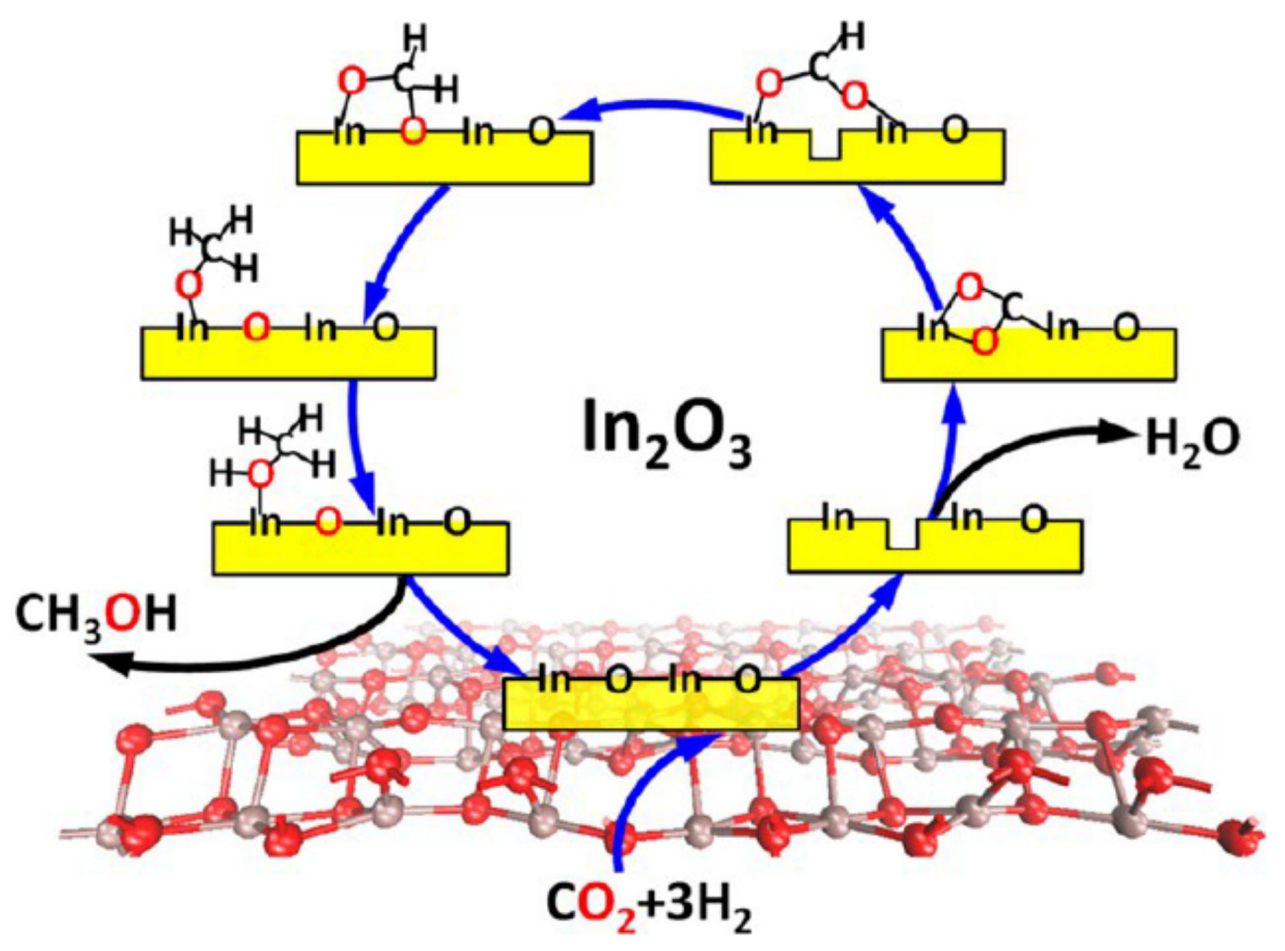
Possible oxygen vacancies formation mechanism and their healing in CO_2_ hydrogenation to methanol. Reproduced with permission from the authors of [152]. Copyright 2013 American Chemical Society.

CO_2_ hydrogenation to methanol on In_2_O_3_ surface with an oxygen vacancy was investigated by Ye et al. [152] using periodic DFT calculations. Different kinds of oxygen vacancies were identified with dissimilar energy states. On the surface with the highest energy, one of the O atoms of the CO_2_ molecule filled in the site upon adsorption. The hydrogenation process that proceeded passed through the HCOO* intermediate. Further hydrogenation of HCOO* formed the C–H bond following the C–O bond scission, resulting in H_2_CO* and hydroxyl. This step was slightly endothermic with a barrier of 0.57 eV but both thermodynamically and kinetically favorable. These observations confirm that the oxygen vacancy on the In_2_O_3_ (110) surface assists CO_2_ activation and hydrogenation and also stabilizes the key intermediates (HCOO*, H_2_COO*, and H_2_CO*) involved in methanol formation. The methanol molecules healed the oxygen vacant sites by replenishing them (Figure 11) with the assistance of H_2_, sustaining the catalytic cycle. Oxygen vacancy catalyzed the formate dissociation to methanol, which was the rate-determining step on Ru/CeO_2_ catalyst. Moreover, the activation temperature for the formate route was much lower than that for the CO route (150 °C vs. 250 °C), which shows the advantage of oxygen vacancy in promoting the rate-determining step [173].

**Figure 12:**
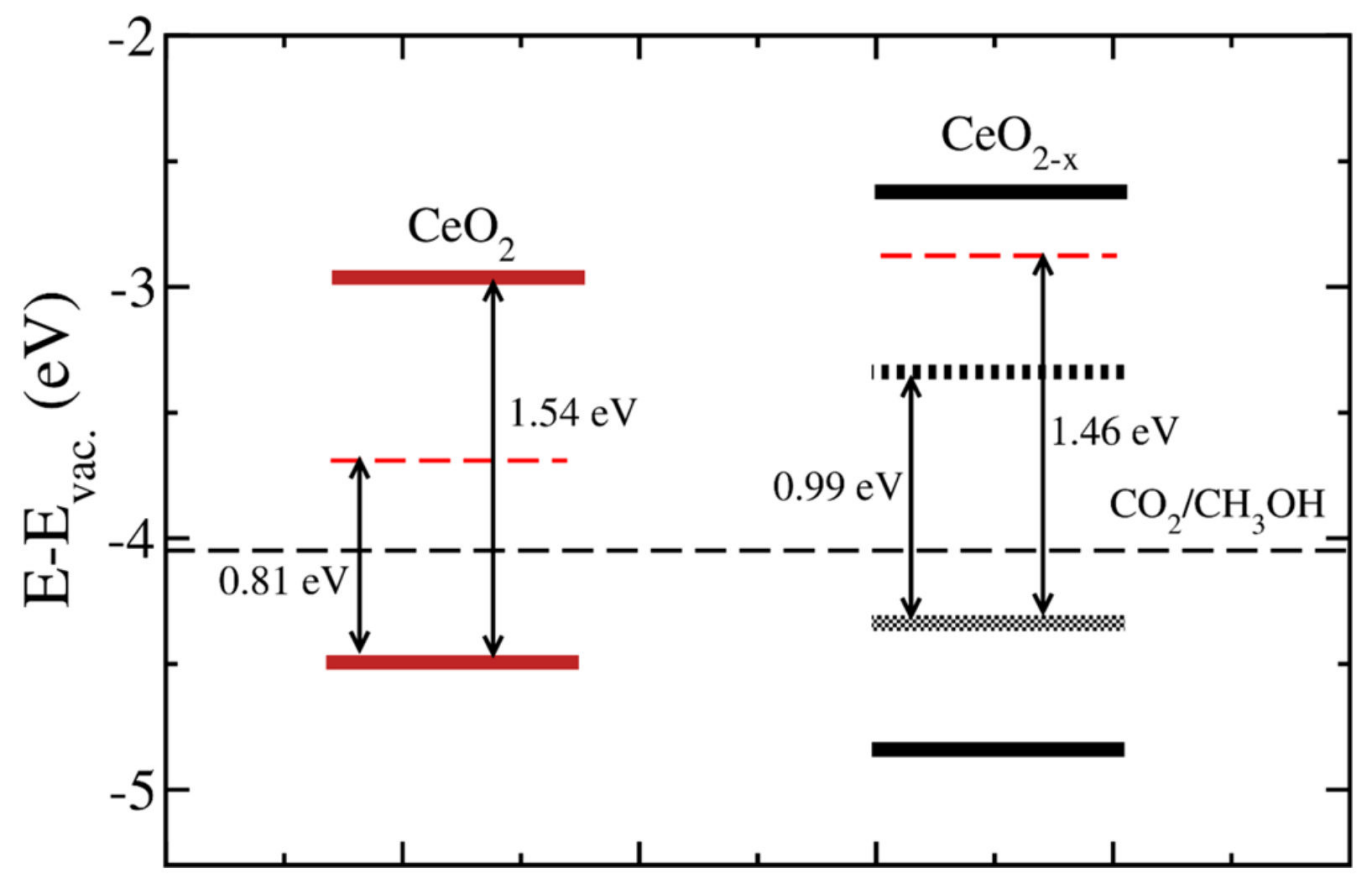
Valence band top and conduction band bottom energies of CeO_2_ surface without (CeO_2_) and with (CeO_2−x_) oxygen vacancies. Modified surface state and defect peak are shown with the shaded and stripped black blocks, respectively. Reproduced with permission from the authors of [119]. Copyright 2019 American Chemical Society.

The introduction of oxygen vacancies into ZnO nanosheets created a new defect level around the valence band maximum where abundant localized electrons accumulated. This formed a new electronic structure that benefited the activation of CO_2_ as revealed by the lower Gibbs free energy and lower energy barrier of the rate-determining step than that of the ZnO slab for the formation and activation of *COOH intermediates [74]. The Bi_2_Sn_2_O_7_ NPs with oxygen vacancies possess the ability to back donate an electron and optimize the electronic structure for effective CO_2_ activation as proven by DFT calculations, revealing through the catalytic performance efficient stabilization of the *COOH intermediates, and energy barrier reduction for CO desorption which was the determining step [164]. Abundant oxygen vacancies in Bi_24_O_31_Cl_10_ improved separation of electron–hole pairs, enhanced charge density, and lowered activation energy for the formation of intermediates during the photocatalytic CO_2_ reduction that led to an optimized CO_2_ conversion efficiency [148]. The photocatalytic performance (enhanced light absorption and improved charge carrier separation) of CeO_2_ nanoparticles (12–18 nm) was improved in the presence of higher concentration of oxygen vacancies, which suppressed the electron–hole recombination as studied using photoluminescence, and confirmed with photocurrent measurement results. The surface oxygen vacancies modified the surface state above the valance band, as seen in the upward shift relative to the vacuum (Figure 12). In the photoelectrochemical CO_2_ reduction system, introducing oxygen vacancies into the lattice of a semiconductor catalyst can alter its intrinsic electronic properties and band gap, promote the transfer of electrons and facilitate adsorption and activation of CO_2_ [139].

#### 3.3.4. The Roles of Solvent in CO_2_ Transformation

There have been interesting attempts to replace water with other solvents as reductants in photocatalytic CO_2_ reduction. For example, as described in a study by Srinivas et al. [174], CO_2_ reduction with CdS in various solvents (water, methanol, ethanol, and 1-propanol of different dielectric constants of 80, 33, 24.3, and 20.1, respectively) was experimentally investigated. Formate and CO were observed as the major products in various solvents deployed as reductants in the photocatalytic reduction of CO_2_ using TiO_2_ nanocrystals embedded in SiO_2_ matrices as the photocatalyst [175]. The characteristics of the solvent (defined with polarity/dielectric constant) and CO_2_ adsorption/dissolution will determine the selectivity toward the product. Based on the polarity and dielectric constant, solvents with low dielectric constant or low polarity could in situ generate CO_2_^−^ anionic radicals that were strongly adsorbed on the surface of the catalyst through the carbon atom because the CO_2_^−^ anion radicals were poorly dissolved in the solutions [174,175]. This will lead to the formation of CO as the major product. In solutions with high dielectric constants, such as water, the CO_2_^−^ anion radicals are well stabilized in the solvent, resulting in a weak interaction with the photocatalyst surface. In this case, the carbon atom tends to be attracted to a proton, exhibiting a high propensity toward formate formation [175]. However, water is often deployed for the photocatalytic (artificial photosynthesis) and, in some cases, electrocatalytic CO_2_ reduction reactions.

The change in the adsorbed surface species and surface chemical state of catalysts has been correlated with the moisture or humidity change during chemical reactions [107]. Adsorbed water takes part in the reaction chemistry of CO_2_ on metal oxide surfaces. Water molecules involve in the CO_2_ reduction process by providing hydroxyl ions and protons that facilitate the electron transfer [176]; thus, H_2_O is beneficial for hydroxylation of the catalyst surface. In addition, the adsorption and dissociation of H_2_O to protons on the catalyst surface can activate deep reduction, leading to highly reduced state hydrocarbon products [32]. On the hydroxylated metal/metal oxide surfaces, CO_2_ can bind as bicarbonates and carbonates in monodentate and bidentate modes [114]. Co-adsorbed water was essential for activating CO_2_ by forming surface adsorbed intermediates during methanol synthesis on metal/oxide catalysts [177]. Most photocatalytic CO_2_ reduction reactions proceed in the presence of H_2_O. Water participates in the reaction by capturing photogenerated holes to generate O_2_, thus reducing the recombination rate of photogenerated holes and electrons. Under this condition, the utilization efficiency of photogenerated electrons can be improved [32]. Although water is often used as the reducing agent in photocatalytic process, pure water is hardly used. The use of NaOH-containing water solution is practiced due to undisputed benefits that include: (i) facilitating the dissolution of adsorbed CO_2_ due to its high affinity with caustic NaOH solution, and (ii) slowing the electron-hole pairs recombination, leading to more utilization of surface electrons that facilitates CO_2_ reduction. The OH ions in aqueous basic solutions act as strong hole scavengers and readily form ^•^OH radicals [174].

In the absence of co-adsorbed H_2_O, the in situ H_2_O formation is possible via the following mechanism. In the presence of H_2_ as a reducing agent, over the surface of supported metals/metal oxides, H_2_ dissociates at the metal sites or metal-oxide interface to adsorbed H* atoms, which then migrate to the active sites and react with the surface oxygen atoms of metal oxide or interact with the adsorbed CO_2_ species in a stepwise fashion to form water [178]. Therefore, the hydroxylation of the catalyst surface is still possible when H_2_ is deployed as a reductant, and the facile and efficient activation of hydrogen is necessary. Using DFT, CO_2_ adsorption was investigated on the In_2_O_3_ (110) surface for methanol synthesis to understand the benefits of H_2_ and H_2_O presence. Results revealed the hydroxylation of the catalyst surface. H_2_ dissociated homolytically on the oxide surface with a high surface coverage, leading to the formation of water. This induced the formation of surface OH-groups, which reduced under-coordinated In atoms at the surface, giving rise to In ion sites and enhanced the formation of oxygen vacancies observed at high hydrogen coverages [179]. However, the oxygen vacancies formed on In_2_O_3_ (110) surfaces did not facilitate CO_2_ adsorption during the reaction. CO_2_ adsorption was facilitated on hydroxylated surface sites, leading to activation by accepting one electron from the In_2_O_3_ (110) surface [179]. Su et al. [112] observed the promotion of a reaction with adsorbed oxygen which formed H_2_O after the introduction of H_2_ during the RWGS, although the adsorbed H_2_O desorbed from the surface at high temperatures. It was also found that adsorbed CO produced more in the presence of H_2_.

**Figure 13:**
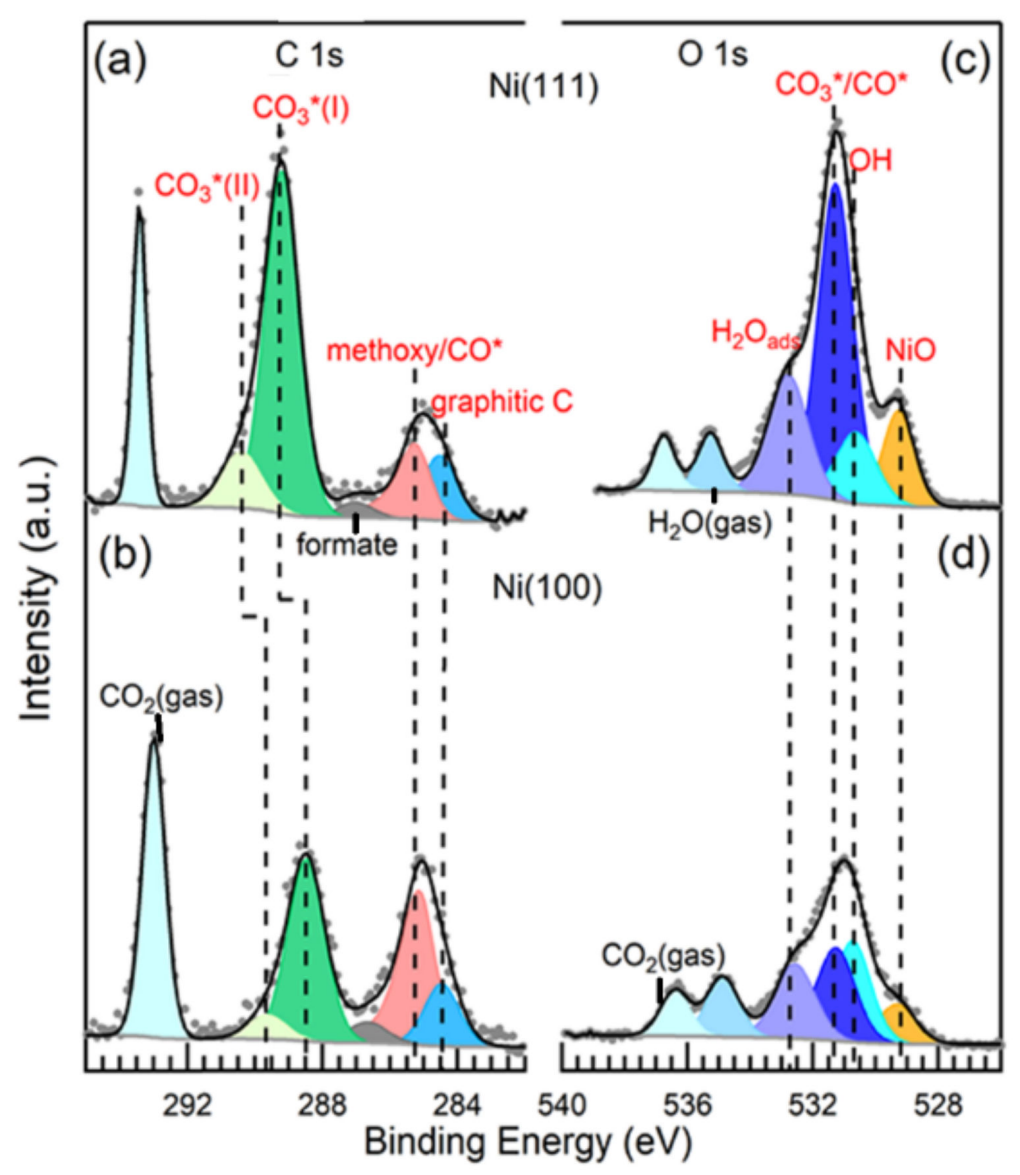
Ambient pressure XPS spectra of (**a**) C 1s, (**c**) O 1s of Ni (111) and (**b**) C 1s, (**d**) O 1s of Ni (100) in 0.2 Torr CO_2_ + 0.2 Torr H_2_O at room temperature. Reproduced with permission from the authors of [107]. Copyright 2019 American Chemical Society.

The effect of H_2_O on Ni surfaces pre-exposed to CO_2_ was investigated by Cai et al. [107]. As observed using ambient air XPS measurements (Figure 13c,d), a new peak evolved in the O 1s spectra at 535.3 eV due to the gas-phase H_2_O, that transformed to surface hydroxyl and adsorbed water with increased concentrations as revealed by the enhanced peak intensities at 530.9 and 532.6 eV, respectively. It obviously indicates that adsorption and dissociation of H_2_O occurred on the Ni surfaces, producing H atom and OH, which further reacted with CO_2_ and other surface species to produce CO_2_ hydrogenation intermediates and product species (formate, methoxy/CO*, and carbonate species) as shown in Figure 13a,b.

The interaction of CO_2_ with a ZrO_2_ model surface (a O–Zr–O trilayer grown on Pt_3_Zr (0001)) with and without the presence of H_2_O was measured by in situ near ambient (atmospheric) pressure XPS (NAP-XPS) and infrared reflection absorption spectroscopy (IRAS). No interaction was observed with the ZrO_2_ model surface at room temperature upon exposure to pure CO_2_ up to 3 × 10^−2^ mbar. Co-adsorption of CO_2_ with H_2_O led to the formation of various carbonaceous surface species. This means that activation of CO_2_ occurred near ambient pressures under the humid condition due to surface hydroxylation that led to the formation of surface species identified by the combined NAP-XPS and IRAS measurements [177]. On reduced CeO_x_-TiO_2_ composite surfaces, the interaction of CO_2_ and H_2_O was thermodynamically favorable at multiple sites. H_2_O molecules were observed to spontaneously dissociate at the oxygen vacant sites, generating surface hydroxyl groups that reacted with CO_2_ to form carbonaceous species—carboxylate or bicarbonate species—according to the NEXAFS results [130]. The apparent correlation between CO_2_ and water was investigated via infrared experiments performed using regular and deuterated water to pre-cover the surface prior to the CO_2_ adsorption. It was found that the degree of hydroxylation of the surface plays a crucial role in CO_2_ adsorption on regular (Fe_tet1_) tetrahedrally terminated magnetite (Fe_3_O_4_) surface, leading to the formation of surface bicarbonates [144]. Surface hydroxide sites in hydroxylated indium oxide (In_2_O_3__−x_(OH)_y_) acted as Lewis base for CO_2_ activation [180]. In the presence of co-adsorbed water, metal oxides (FeO_x_, Al_2_O_3_, and TiO_2_) exhibited high reaction rates for CO_2_ adsorption under ambient conditions and, thus, can improve the CO_2_ adsorption kinetics [176].

Although the positive effects of moisture have been discussed, an experimental report revealed a 20% decrease in methanol yield under standard methanol synthesis conditions when water was co-fed into an H_2_/CO_2_ mixture over In_2_O_3_ surface [181]. The co-adsorption of H_2_O suppressed the interfacial interaction between CO_2_ and Ni surface [107]. On defective β-Ga_2_O_3_ (100) with rich oxygen vacancy, water spontaneously enhanced dissociative adsorption of CO_2_ at the oxygen vacancy site, with reaction energy of −0.62 eV. These results indicate that the co-existence of water and CO_2_ in the adsorption system imposes competition with CO_2_ for the oxygen vacancy sites, which negatively impacts CO_2_ chemisorption and conversion [151].

#### 3.3.5. General Methods for Modifying Metal Oxide Surface Structure for CO_2_ Transformation

Several approaches can be applied to modify the structure of metal oxides for the facile adsorption and dissociation of CO_2_ molecules, mainly, through tailoring their physicochemical (e.g., improving surface basicity) [182,183] and electronic properties (e.g., doping and creating defect sites) [182].

(i) Insertion of acidic and basic surface sites. The basic sites on the catalyst surface can facilitate CO_2_ adsorption because CO_2_ is an acidic compound whose interaction with the catalyst surface will be enhanced with a basic surface. The basic sites of metal oxide catalysts can be generated by pre-treatment with basic promoters such as La_2_O_3_, K_2_O, Na_2_O, and MgO [183]. Tian et al. [182] reported that introducing potassium into iron oxide catalyst increased the surface basicity. Thus, alkali metals can be incorporated into the metal oxides to tune the concentration of surface basic sites. The basic sites enhance the activation of acidic CO_2_ and also help to limit carbon deposition. The overall benefit is improvement in catalytic activity and stability. For example, in the CO_2_ dry reforming of methane, the catalytic reaction was initiated by an acid–base interaction [183]. On the In_2_O_3__−x_(OH)_y_ surface, comprising frustrated Lewis pairs (FLP) (A frustrated Lewis pairs consists of both Lewis acid and Lewis base that cannot combine to form an adduct due to steric hindrance.), the surface hydroxide site acts as Lewis base and the coordinately unsaturated In surface site acts as a Lewis acid to activate CO_2_ [180]. The Lewis acid and Lewis base synergistically interact to heterolytically dissociate H_2_ adsorbed on the In_2_O_3−x_(OH)_y_ surface, forming protonic surface FLP sites that can capture and convert CO_2_ to CO and H_2_O. The surface Lewis basicity can be tuned by the nature of the metal site, which can be controlled by the size, charge, coordination number, geometry, and electronegativity of the metal in a particular oxidation state [114]. Surface basic sites can be characterized using CO_2_-TPD. Typically, desorption at high temperatures signifies the presence of strong basic sites. The CO_2_ adsorption strength on metal oxides has been related to the basicity, improving as the concentration increases. However, too strong adsorption may result in the formation of undesirable intermediate species such as surface carbonate and bicarbonate species.
(ii) Metal oxide doping. Doping metal oxides with metallic elements can optimize their electronic and geometric structures and enhance catalytic performance. Alkali metals doping can modify the electronic structure by increasing the concentration of electron-withdrawing groups such as surface hydroxyl groups or reducing the kinetic barrier for CO_2_ dissociation [184]. Moreover, oxides with electron-rich defect centers may interact readily with CO_2_ by donating an electron. These, in turn, will facilitate surface reaction activity, boosting catalytic efficiency for CO_2_ activation. The list is not exclusive to alkali metals, as some transition metals have the potentials to tune the electronic characteristics of the doped oxide. Doping transition metal oxide to the based oxide can as well tune the concentration of oxygen vacancies
(iii) Defect engineering. It involves creating or adjusting surface oxygen vacancies or hydroxyl groups in an oxide. The presence of oxygen defective sites can similarly impact the surface electronic property. Evidence has linked the activity of an oxide catalyst with surface defects. For example, it was demonstrated that the activity of MnO_2_ in HCHO oxidation correlated with the concentration of surface defects [185]. CeO_2_ nanoparticles with a high concentration of oxygen vacancies had high photocatalytic performance in converting CO_2_ to methanol (0.702 μmol h^−1^ g^−1^) [119]. Furthermore, oxygen vacancies are sites for adsorption and anchoring of CO_2_ molecules. Consequently, surface adsorption and reactivity of adsorbates, including H_2_ and H_2_O, would be facilitated in the presence of extra electrons in the vicinity of oxygen vacancies. The amount of defective sites in a metal oxide can be controlled by oxidative treatment during the catalyst synthesis and catalytic reactions.
(iv) Generation of surface hydroxyls. The surface hydroxyl groups participate in CO_2_ hydrogenation by incorporating hydrogen atom into CO_2_, facilitating CO_2_ activation and accelerating reaction rate. Mechanistic studies evidenced the surface hydroxyl species on SiC quantum dots (QDs) to directly participate in CO_2_ hydrogenation through the addition of H atoms of hydroxyl groups into CO_2_ to form HCOO* [186]. The surface hydroxide site on the In_2_O_3__−x_(OH)_y_ surface acted as a Lewis base that interacted with CO_2_ [180].
(v) Mixed metal oxides or composites catalysts can yield good catalytic performances. Introducing a second oxide component can improve adsorption, increase the effective oxygen vacancies, and present an interfacial surface for reactions (adsorption/activation and desorption). When preparing a mixed-metal oxide catalyst, consideration should be given to factors that can improve catalytic performance, such as elemental composition since catalytic activity can be a function of how elements in the composite are combined. The deposition of sub-monolayer amounts of a second oxide over a host oxide can create nanostructures that can enhance the overall catalytic properties of the composite system [114,187].
(vi) Forming metallic–non-metallic hybrid catalysts. These kinds of catalysts have high surface areas and improved adsorption properties. Carbon materials, such as carbon nanotubes (CNTs) and graphene oxides (GOs), and metal oxides (e.g., TiO_2_, CeO_2_, Al_2_O_3_, ZrO_2_, In_2_O_3_, Ga_2_O_3_, and NiO) can be grafted to form hybrid nanostructured materials [154,188,189,190]. Carbon materials possess very high surface areas, whereas metal oxides are endowed with rich oxygen vacancies. Combining materials with these features can result in a unique hybrid material with superior catalytic performance toward CO_2_ reduction reactions. The high surface area will enhance adsorption complemented by oxygen vacancies, which are also sites for CO_2_ activation on oxide catalysts. Modification of CuO–ZnO–ZrO_2_ with GO increased the adsorption capacities of both CO_2_ and H_2_, resulting in an improved catalytic activity (methanol selectivity) in methanol synthesis than the GO-free catalyst [188]. The electronic structure of the traditional methanol synthesis catalyst (Cu–ZnO–Al_2_O_3_) was altered by adding the N-doped graphene, which provided a synergistic effect for methanol synthesis [189]. Incorporating metal oxides to single-wall CNT electrode can form a hybrid structure with an increase in specific surface area, which also improved electrical conductivity and charge transfer [191,192]. Moreover, doping carbon into photocatalysts can enhance light absorption and improve the photothermal conversion efficiency by reducing the energy for oxygen vacancy formation, thus generating a high concentration of active sites [190]. Therefore, hybrid nanostructured materials with good electronic and charge transfer properties can be explored for both thermochemical and electrochemical reduction of CO_2_.
(vii) Stabilization of metal oxide surface. Reduced oxides are more susceptible to react with CO_2_ [118,193]. Stabilizing the reduced surface can be achieved by creating a conducive environment for reactions such as forming oxide–metal or oxide–oxide interfaces [128,187,194]. The synergistic properties associated with these interfaces can improve the surface chemistry of metal oxides and could be beneficial for synthesizing efficient catalysts for CO_2_ transformation.

## 4. Conclusions

Controlling the emission of CO_2_ by converting it to products of economic value is highly attractive. However, CO_2_ is a kinetically stable molecule that requires high energy input for the C–O bond breaking. Its proper activation can reduce the high energy barrier substantially, easing conversion by various processes. The CO_2_ activation is an important step that precedes the conversion of CO_2_ to chemicals and fuels. It can be effected in the presence of a catalyst by decreasing the O–C–O angle from 180 degrees (bending), weakening and elongation of the C–O bonds, polarizing the charges on C and O by charge/electron transfer, and redistribution of charges on the CO_2_ molecule. Upon accepting an extra electron from a substrate, the neutral CO_2_ molecule forms an anion with a full charge (CO_2_^−^) or partial charge (CO_2_^δ−^). However, a recent study has proposed that just a charge transfer is insufficient to activate CO_2_ molecules on metal oxide catalysts, but the binding of an O atom to a surface metal cation weakens and elongates the molecular C–O bond.

Some metals and metal oxides are efficient catalysts for CO_2_ conversion reactions; thus, they should be good for CO_2_ activation. In general, metal nanoparticles serve as active sites for electron transfer, with certain factors such as change in morphology of metal particles, nanoparticle size, adsorption mode and configuration, and chemical ordering as the CO_2_ activation marker. The interaction of CO_2_ with pure metals is rather weak but can be improved by incorporating promoters (e.g., alkali metals) with low electronegativity. Metal oxide nanoparticles are utilized as supports or directly as catalysts for the CO_2_ conversion. Their surfaces comprise both metal (M^n+^) and oxygen (O^2−^) ions, which can act as active sites for CO_2_ activation. They can activate CO_2_ by coordinating to one or two adjacent metal sites through the terminal oxygen atoms of the CO_2_ or interaction of the carbon atom of CO_2_ with surface oxygen sites. A particularly interesting feature in metal oxides is the oxygen vacancies.

Oxygen vacancies are of great benefits to the overall reaction thermodynamics and kinetics and can greatly enhance the adsorption energy of CO_2_ molecules, promoting interaction of CO_2_ with the oxide surface. Indeed, oxygen vacancies play vital roles in the overall CO_2_ conversion process, ranging from electronic structure modification, selection of reaction pathway, and stabilization of key intermediates to the creation of binding surface, sites for CO_2_ adsorption, and dissociation. Although being an integral part of reducible oxides, the concentration of oxygen vacancies can be increased or created in metal oxide catalysts either in situ during reactions or incorporated by external methods, including thermal annealing at high temperatures, doping, plasma-assisted techniques, photo-irradiation, and solid-state chemical reactions during synthesis or by post-synthesis techniques.

The surface hydroxyl groups, which can be introduced in metal oxide catalysts in the presence of moisture, also present significant CO_2_ activation enhancement by facilitating interaction and providing additional binding sites.

Several strategies are applicable to modify metal oxide catalysts for CO_2_ activation and conversion. Creating more basic surface sites can stimulate surface reaction with acidic CO_2_. Other strategies include elemental doping and defect engineering. Surface defect sites are good adsorption sites for CO_2_ on metal oxides. Doping with low electronegative elements can enrich the surface with electrons to easily transfer to CO_2_ upon adsorption. Like the case of basic sites, generating more hydroxyl groups to make the surface hydrophilic can boost the catalytic performance of metal oxides, similar to those of metal hydroxides or layered double hydroxides. Hybrid catalysts, such as metal oxide/carbon material and composite oxide catalysts, tend to improve the performance of the resulting system through the synergism of the individual components. In these processes, it is possible to stabilize the metal surface by forming oxide–metal or oxide–oxide interfaces. The synergetic properties associated with such interfaces can improve the surface chemistry for reactions. In view of the catalysts discussed in this work, metal oxides with large numbers of oxygen vacancies exhibit excellent performance for CO_2_ adsorption and activation. In the present, researchers have demonstrated the ability of pure (e.g., In_2_O_3_) or doped (ZnO-ZrO_2_) metal oxides and metal–organic framework to reduce CO_2_ to methanol, and of course, some other products. However, challenges remain due to the relatively low catalytic activity and selectivity of these materials. Perhaps, their bulk forms are presented with these limitations. Furthermore, other factors, such as the strong metal-support interactions (SMSI) for supported metal catalysts on the reducible oxide supports, should be considered, as the reduced support oxide can migrate and cover the active metal particles, significantly impacting the electronic and interface structure of the catalysts [195,196].

Specially designing and engineering surface functionalities in these materials, including inserting a controllable amount of oxygen vacancies in the right proportion and surface hydroxyls, is capable of tuning the catalytic properties, in which activation of CO_2_ molecule should be a topmost consideration. The future offers great opportunities to develop even more active heterogeneous catalysts for the various CO_2_ reduction reactions. The possible structural transformations of the catalysts, which remain elusive, can be unveiled to address the complexities involved in these reactions fully. Identifying the catalyst structural defects, their formation or transformation during the reaction, and their contribution to the overall activity is still a challenging task, yet of significant importance. Theoretical models can provide predictions on the catalyst system, and such methods should be deployed to model new metal oxide catalysts with incorporated functionalities.

## Figures and Tables

**Table 1:** Standard enthalpies (ΔH°_298K_) and Gibbs free energies (ΔG°_298K_) for different products formed via CO_2_.

Equation	Reaction	ΔH° _298 K_ (kJ·mol^−1^)	ΔG° _298 K_ (kJ·mol^−1^)
(1)	$2{\mathrm{CO}}_{2}\to 2\mathrm{CO}+O_{2}$	293.0	257.2
(2)	${\mathrm{CO}}_{2}+H_{2}\to \mathrm{CO}+O_{2}$	41.2	28.6
(3)	${\mathrm{CO}}_{2}+3H_{2}\to {\mathrm{CH}}_{3}\mathrm{OH}+H_{2}O$	−49.5	3.5
(4)	$2{\mathrm{CO}}_{2}+6H_{2}\to 2C_{2}H_{5}\mathrm{OH}+3H_{2}O$	−86.7	−32.4
(5)	${\mathrm{CO}}_{2}+4H_{2}\to {\mathrm{CH}}_{4}+2H_{2}O$	−165.0	−113.5
(6)	$2{\mathrm{CO}}_{2}+7H_{2}\to C_{2}H_{6}+4H_{2}O$	−132.1	−78.7
(7)	$3{\mathrm{CO}}_{2}+10H_{2}\to C_{3}H_{8}+6H_{2}O$	−125.0	−70.9
(8)	$4{\mathrm{CO}}_{2}+13H_{2}\to n-C_{4}H_{10}+8H_{2}O$	−121.6	−66.9
(9)	$2{\mathrm{CO}}_{2}+6H_{2}\to C_{2}H_{4}+4H_{2}O$	−64.0	−28.7
(10)	$3{\mathrm{CO}}_{2}+9H_{2}\to C_{3}H_{6}+6H_{2}O$	−83.6	−42.1
(11)	$4{\mathrm{CO}}_{2}+13H_{2}\to n-C_{4}H_{8}+8H_{2}O$	−90.3	−45.2

Thermodynamics data adapted with permission from the authors of [17]. Copyright 2016 Elsevier B.V.

**Table 2:** Properties of CO_2_ molecule.

Property	Boiling Point (° C)	Density Gas (g/L)	Dipole Moment	Bond Polarity	∆H_f_ (_298 K_) (kJ·mol^−1^)	Bond Energy (kJ·mol^−1^)	C–O Bond Distance (Å)	Bond Angle (degree)	Band Gap	Charge	^a^ HOMO Structure	^b^ LUMO Structure
CO_2_	−78.5 (sublimes)	1.98	0	1.0	−393.5	806	1.16	180	ca. 8 e	C: −0.360 O: +719	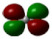	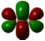

^a^ Highest occupied molecular orbital, ^b^ Lowest unoccupied molecular orbital (high energy for ground state CO_2_ molecule).

**Table 3:** DFT-calculated adsorption energies, geometries, and charge transferred to CO_2_ on single crystal surfaces of metals. Data extracted with permission from the authors of [88]. Copyright 2016 American Chemical Society.

Metal	Physisorption	Chemisorption		
	Binding Energy (kJ·mol^−1^)	OCO Angle/°	Binding Energy (kJ·mol^−1^)	Net Charge/e
Fe (110)	−23	121	−90	−1.11
Ir (111)	−33	128	−34	−0.47
Pd (111)	−32	140	−17	−0.35
Ru (0001)	−31	123	−61	−0.83
Rh (111)	−32	135	−35	−0.46
Ni (111)	−26	136	−20	−0.50
Co (0001)	−25	139	−30	−0.64
Pt (111)	−21	131	−3	−0.36

## Data Availability

The data presented in this study are available on request from the corresponding author.
